# Genome-wide enhancer annotations differ significantly in genomic distribution, evolution, and function

**DOI:** 10.1186/s12864-019-5779-x

**Published:** 2019-06-20

**Authors:** Mary Lauren Benton, Sai Charan Talipineni, Dennis Kostka, John A. Capra

**Affiliations:** 1 Department of Biomedical Informatics, Vanderbilt University, Nashville, TN 37235 USA; 2 Department of Developmental Biology, University of Pittsburgh School of Medicine, Pittsburgh, PA 15201 USA; 3 Department of Computational & Systems Biology, Pittsburgh Center for Evolutionary Biology and Medicine, University of Pittsburgh School of Medicine, Pittsburgh, PA 15201 USA; 4 Departments of Biological Sciences and Computer Science, Vanderbilt Genetics Institute, Center for Structural Biology, Vanderbilt University, Nashville, TN 37235 USA

**Keywords:** Enhancer identification, Gene regulation, Cis-regulatory elements

## Abstract

**Background:**

Non-coding gene regulatory enhancers are essential to transcription in mammalian cells. As a result, a large variety of experimental and computational strategies have been developed to identify *cis*-regulatory enhancer sequences. Given the differences in the biological signals assayed, some variation in the enhancers identified by different methods is expected; however, the concordance of enhancers identified by different methods has not been comprehensively evaluated. This is critically needed, since in practice, most studies consider enhancers identified by only a single method. Here, we compare enhancer sets from eleven representative strategies in four biological contexts.

**Results:**

All sets we evaluated overlap significantly more than expected by chance; however, there is significant dissimilarity in their genomic, evolutionary, and functional characteristics, both at the element and base-pair level, within each context. The disagreement is sufficient to influence interpretation of candidate SNPs from GWAS studies, and to lead to disparate conclusions about enhancer and disease mechanisms. Most regions identified as enhancers are supported by only one method, and we find limited evidence that regions identified by multiple methods are better candidates than those identified by a single method. As a result, we cannot recommend the use of any single enhancer identification strategy in all settings.

**Conclusions:**

Our results highlight the inherent complexity of enhancer biology and identify an important challenge to mapping the genetic architecture of complex disease. Greater appreciation of how the diverse enhancer identification strategies in use today relate to the dynamic activity of gene regulatory regions is needed to enable robust and reproducible results.

**Electronic supplementary material:**

The online version of this article (10.1186/s12864-019-5779-x) contains supplementary material, which is available to authorized users.

## Background

Enhancers are typically defined as genomic sequences that regulate the transcription of one or more genes, regardless of orientation or relative distance to the target promoter [1]. These *cis*-regulatory regions can bind specific transcription factors and cofactors to increase transcription, and in current models of enhancer function, they physically interact with their long-range targets via loops in the three-dimensional chromatin structure [1–3]. Enhancers play a vital role in the regulation of genes during development and cell differentiation [3, 4], and genetic variation in enhancers has been implicated in etiology of complex disease [5, 6] and in differences between closely related species [7–9].

Given their importance, enhancers have seen considerable study in recent years. More than 2300 papers have been published on enhancer biology (MeSH: *Enhancer Elements, Genetic*) since the start of 2015, and hundreds of these have focused on the role of enhancers in disease. Despite the importance of enhancers, they remain difficult to identify [1, 10, 11]. Experimental assays that directly confirm enhancer activity are time-consuming, expensive, and not always conclusive [1, 12]. And, despite recent promising developments in massively parallel reporter assays (MPRAs), current methods are unable to definitively identify and test enhancers on an unbiased genome-wide scale [13]. As a result, many studies have used more easily measurable attributes associated with enhancer activity, including DNA sequence motifs, evolutionary conservation, and biochemical modifications, as proxies for enhancer activity [1, 13–16]. For example, active enhancers localize in regions of open chromatin, which are commonly assayed by testing the sensitivity of DNA segments to DNase I nuclease followed by sequencing (DNase-seq) [17, 18]. Enhancers also often have characteristic sets of histone modifications on surrounding nucleosomes. Monomethylation of lysine 4 on histone H3 (H3K4me1) and lack of trimethylation of lysine 4 on histone H3 (H3K4me3) are used to distinguish enhancers from promoters, while acetylation of lysine 27 on histone H3 (H3K27ac) often denotes active enhancers [1, 14, 19]. Additionally, features such as evolutionary sequence conservation [20–22] and the presence of known transcription factor binding motifs [16] or known enhancer associated proteins, such as the histone acetyltransferase p300 [14, 23, 24], have been used to successfully locate enhancer elements [1]. Furthermore some enhancers are transcribed, and active enhancers can be mapped by identifying characteristic bi-directionally transcribed enhancer RNAs (eRNAs) [25, 26], although the specificity of this pattern to enhancers has recently been questioned [27]. While informative, none of these attributes are comprehensive, exclusive to enhancers, or completely reliable indicators of enhancer activity. In addition, enhancer activity is also context- and stimulus-dependent, creating a further layer of complexity [19, 28].

In addition to these experimental approaches, many complementary computational enhancer identification methods have been developed that integrate the above-mentioned data in both supervised and unsupervised machine learning approaches [11, 15, 29]. Since there are no genome-wide gold standard enhancer sets, such enhancer identification studies and algorithms validate their results via a combination of small-scale transgenic reporter gene assays and enrichment for other functional attributes, such as trait-associated genetic variants, evolutionary conservation, or proximity to relevant genes. As a result, there is substantial variation in these computational approaches [11].

The genome-wide enhancer maps resulting from these experimental and computational approaches are commonly used in many different applications, including studies of the gene regulatory architecture of different tissues and the interpretation of variants identified in genome-wide association studies (GWAS) [5, 6, 30]. However, in these applications, a single enhancer set frequently serves as the working definition of an “enhancer” for all analyses and individuals. Furthermore, a recent study found significant discordance in regions identified by histone marks plus ChromHMM predictions and activity levels in an MPRA [31].

Here, we evaluate the robustness of the “single definition” approach by performing a comprehensive analysis of similarities in genomic, evolutionary, and functional attributes of enhancers identified by different strategies in two tissues (liver and heart) and two cell lines (K562 and Gm12878). By comparing characteristics of different enhancer sets identified in the same biological context, we were able to assess the stability of conclusions made using only one enhancer identification strategy. All methods overlapped more than expected by chance, but we observed striking differences between the strategies we compared. While some variation is expected due to differences in the underlying assays, the differences between identification strategies applied within the same context are substantial enough to influence biological interpretations and conclusions about enhancer evolution and disease-associated variant function. In our comparisons, we find that enhancers defined by transcriptional signatures, such cap analysis of gene expression or global run-on sequencing, are modestly more enriched for proxies of function than those identified based on evolutionary or functional genomics data; however, they identify a much smaller fraction of active enhancers than other approaches. We also demonstrate that focusing on enhancers supported by multiple identification methods does not provide a satisfactory remedy for disagreement between methods, and it ignores many functional enhancers. As a result, we cannot recommend the use of any single current enhancer identification strategy in isolation or simply considering the intersection of many strategies. These results highlight a fundamental challenge to studying gene regulatory mechanisms and to evaluating the functional relevance of thousands of non-coding variants associated with traits. Incorporating the unique characteristics of different enhancer identification strategies will be essential to ensuring reproducible results and to furthering our understanding of enhancer biology. As a step toward this goal, we have created creDB, a large, easily queried database of putative enhancers identified by different methods across common tissues and cellular contexts.

## Results

### A panel of enhancer identification strategies across four biological contexts

**Fig. 1:**
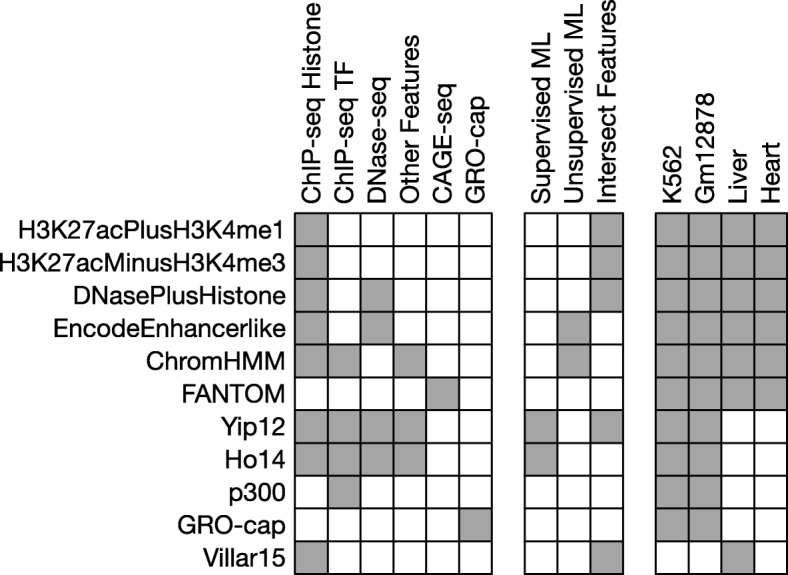
Eleven diverse enhancer identification strategies were evaluated across four cellular contexts. Each row summarizes the data sources, analytical approaches, and contexts for the eleven enhancer identification strategies we considered. The leftmost columns of the schematic represent the experimental assays and sources of the data used by each identification strategy. The middle columns describe the computational processing (if any) performed on the raw data (ML: machine learning). The rightmost columns give the contexts in which the sets were available. Table 1 gives the number, length, and genomic coverage of each enhancer set

To evaluate the variation in enhancer sets generated by different enhancer identification strategies, we developed a consistent computational pipeline to compare enhancer sets genome-wide. Our approach is based on publicly available data, and we applied it to a representative set of methods in four common cell types and tissues (biological contexts): K562, Gm12878, liver, and heart cells (Fig. 1 and Methods). Given the large number of enhancer identification strategies that have been proposed [1, 11], it is not possible to compare them all; so for each context, we consider methods that represent the diversity of experimental and computational approaches in common use.

For all contexts, we consider two enhancer sets derived solely from chromatin immunoprecipitation followed by sequencing (ChIP-seq) for histone modifications informative about enhancer activity from the ENCODE Project [32]. The “H3K27acPlusH3K4me1” set includes all H3K27ac ChIP-seq peaks that also overlap an H3K4me1 peak, and the “H3K27acMinusH3K4me3” set contains H3K27ac peaks that do not overlap an H3K4me3 peak [33–35]. We used broad peak files processed using a consistent custom pipeline and quality control criteria by ENCODE (Methods). In liver only, we consider an additional set of enhancers identified using the H3K27acMinusH3K4me3 definition on different samples (“Villar15”) [35]. We also consider a method that incorporates DNase I hypersensitive sites (DHSs) with histone modifications to generate the “DNasePlusHistone” enhancer set, which is composed of DHSs where the ratio of H3K4me1 to H3K4me3 is less than one [36]. For the two cell lines we also include ChIP-seq peaks for the transcription cofactor p300, “p300”, that is known to be associated with active enhancers [23, 32, 34]. Since transcriptional signatures are increasingly used to identify enhancers, we consider “FANTOM” enhancers identified from bidirectionally transcribed eRNA detected via cap analysis of gene expression (CAGE) by the FANTOM5 Project [25, 37, 38]. For K562 and Gm12878 we include a set of transcribed regions defined by nascent bidirectional transcription in a modification of the global run-on sequencing (GRO-seq) as “GRO-cap” [39]. Finally, we also include several methods that combine machine learning with functional genomics data, such as the ENCODE consortium’s “EncodeEnhancerlike” made by combining DHSs and H3K27ac peaks using an unsupervised ranking method and the “ChromHMM” predictions generated by a hidden Markov model trained on ChIP-seq data from eight histone modifications, CTCF, and RNA Pol II [15, 40–42]. For the K562 and Gm12878 cell lines we include enhancer predictions made by two supervised machine learning methods trained to identify enhancers based on ChIP-seq data in conjunction with other functional genomic features. We will refer to these sets as “Yip12” and “Ho14” [43, 44]. An overview of the data and computational approaches used by each method is given in Fig. 1 and full details are available in the Methods.

### Enhancers identified by different strategies in the same context differ substantially

Each of the enhancer identification strategies considered here is based on molecular signatures resulting from different aspects of the complex processes underlying the regulation of gene expression (e.g., co-factor binding, histone modification, enhancer RNAs). Thus, we anticipated some variation between them; however, given that the results of all of these methods are commonly treated as “enhancers” in practice, we believe that it is valuable to quantify their attributes.

#### The genomic coverage of different enhancer sets varies by several orders of magnitude

**Fig. 2:**
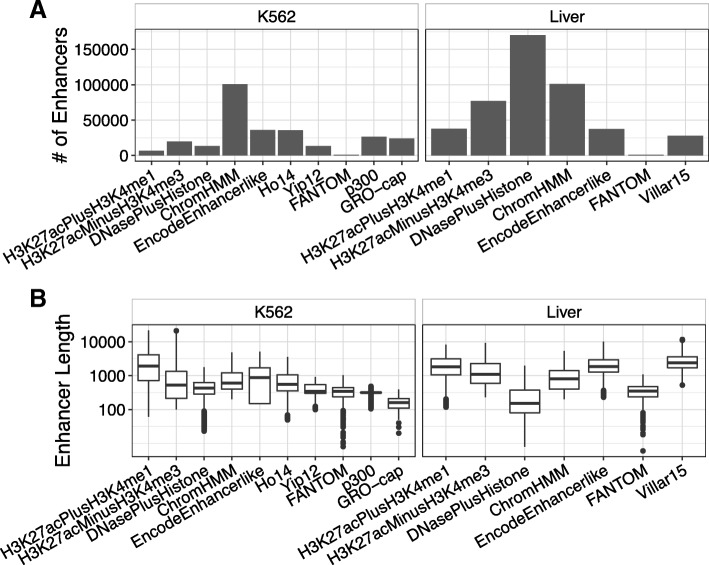
Enhancer identification methods vary in the number and length of predicted enhancers. (**a**) The number of K562 and liver enhancers identified by each method varies over two orders of magnitude. There is considerable variation even among methods defined based on similar input data, e.g., histone modifications. (**b**) The length of K562 and liver enhancers identified by different methods shows similar variation. Enhancer lengths are plotted on a log_10_ scale on the y-axis. Data for other contexts are available in Table 1 and Additional file 1: Figure S1

Enhancer regions identified in the same context by different methods differ drastically in the number of enhancers identified, their genomic locations, their lengths, and their coverage of the genome (Table 1; Fig. 2; Additional file 1: Figure S1). As noted above, we expected to find variation between enhancer sets in these attributes. Nevertheless, the magnitude of differences we observed is striking. For each attribute we considered, enhancer sets differ by several orders of magnitude (Table 1; Fig. 2). For instance, FANTOM identifies 326 kilobases (kb) of sequence with liver enhancer activity, EncodeEnhancerlike identifies 89 megabases (Mb), and H3K27acMinusH3K4me3 identifies almost 138 megabases (Mb).

**Table 1:** Summary of all enhancer sets analyzed in this study

Context	Enhancer Set	Number of Base Pairs (kb)	Number of Enhancers	Median Length	Genome Coverage
K562	H3K27acPlusH3K4me1	22,113	6642	1903	0.0078
	H3K27acMinusH3K4me3	34,072	19,698	525	0.0120
	DNasePlusHistone	6620	13,402	431	0.0023
	ChromHMM	96,545	100,837	600	0.0339
	EncodeEnhancerlike	39,961	36,008	878	0.0140
	Ho14	29,027	35,769	556	0.0102
	Yip12	5389	13,303	342	0.0019
	p300	7939	26,463	316	0.0028
	GRO-cap	3905	23,825	160	0.0014
	FANTOM	390	1084	344	0.0001
Gm12878	H3K27acPlusH3K4me1	28,355	8019	2749	0.0099
	H3K27acMinusH3K4me3	20,868	11,238	701	0.0073
	DNasePlusHistone	9286	19,815	386	0.0033
	ChromHMM	73,929	69,314	800	0.0259
	EncodeEnhancerlike	50,224	38,872	1018	0.0176
	Ho14	41,543	39,550	674	0.0146
	Yip12	5389	13,303	342	0.0019
	p300	6480	17,532	360	0.0023
	GRO-cap	3646	21,308	160	0.0013
	FANTOM	1025	2826	343	0.0004
Liver	H3K27acPlusH3K4me1	87,576	37,644	1831	0.0307
	H3K27acMinusH3K4me3	137,874	77,014	1096	0.0484
	DNasePlusHistone	51,292	170,212	152	0.0180
	ChromHMM	108,375	101,260	800	0.0380
	EncodeEnhancerlike	89,129	37,426	1849	0.0313
	FANTOM	326	869	347	0.0001
	Villar15	86,139	27,725	2545	0.0302
Heart	H3K27acPlusH3K4me1	59,892	42,910	1102	0.0210
	H3K27acMinusH3K4me3	157,468	141,162	684	0.0553
	DNasePlusHistone	33,224	103,898	168	0.0117
	ChromHMM	93,067	113,092	600	0.0327
	EncodeEnhancerlike	186,866	47,235	2872	0.0656
	FANTOM	611	1720	335	0.0002

In addition, methods based on similar approaches often differ substantially due to technical factors; e.g., Villar15, which uses the same enhancer definition as H3K27acMinusH3K4me3, only annotates 86.1 Mb with enhancer function in liver. Enhancer sets also vary in their relative distance to other functional genomic features, such as transcription start sites (TSSs). For example, in liver, the average distance to the nearest TSS ranges from 14 kb for EncodeEnhancerlike to 64 kb for DNasePlusHistone (Additional file 2: Table S1). Overall, as expected, methods based on histone modifications tend to identify larger numbers of longer enhancers compared with CAGE data, and machine learning methods are variable. However, these differences span orders of magnitude. We highlight these trends in liver, but they are similar in other contexts (Table 1; Fig. 2; Additional file 1 :Figure S1).

#### Enhancer sets overlap more than expected by chance but have low genomic similarity

**Fig. 3:**
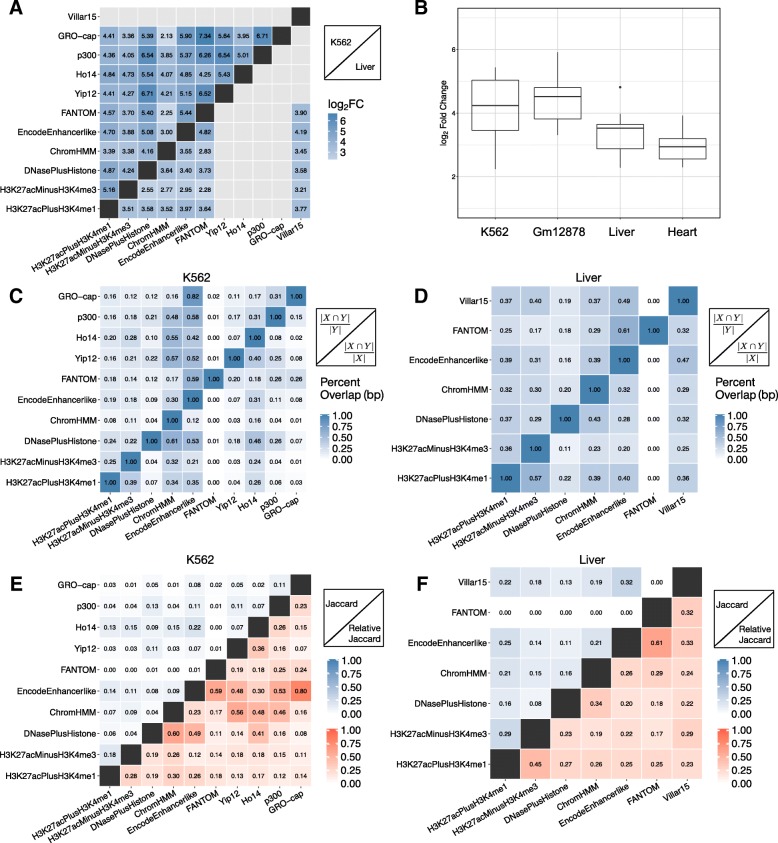
Enhancer sets have low genomic overlap. (**a**) Pairwise bp enrichment values (log_2_ fold change) for overlap between each K562 (upper triangle) or liver (lower triangle) enhancer set, compared to the expected overlap between randomly distributed, length-matched regions. (**b**) The log_2_ enrichment for bp overlap compared to a random genomic distribution for each pair of enhancer sets within each context. Only contexts with annotations across all biological contexts are included. The fold changes across annotations for the primary tissues—liver and heart—are significantly lower than the cell lines—K562 and Gm12878 (*p* = 6.88E-11 Kruskal-Wallis test, followed by Dunn’s test with Bonferroni correction for pairwise comparisons between contexts). The patterns are similar for element-wise comparisons (Figs. S3). (**c**, **d**) The percent base pair (bp) overlap between all pairs of (**c**) K562 enhancer sets and (**d**) liver enhancer sets. Percent overlap for each pair was calculated by dividing the number of shared bp between the two sets by the total number of base pairs of the set on the y-axis. The highest overlap is observed for pairs based on similar input, e.g., machine learning models trained on the same functional genomics data, or comparisons with large sets, e.g. ChromHMM. Comparisons between biological replicates average 76% overlap. (**e**, **f**) The Jaccard similarity between all pairs of (E) K562 or (**f**) liver enhancer sets. The upper triangle gives the Jaccard similarity, and the lower triangle gives the relative Jaccard similarity in which the observed similarity is divided by the maximum possible similarity for the pair of sets

Given the diversity of the enhancer sets identified by different methods, we evaluated the extent of both bp and element-wise overlap between them. All pairs of enhancer sets overlap more than expected if they were randomly distributed across the genome (Fig. 3a,b, ~ 5–100x, *p* < 0.001 for all pairs, permutation test (Methods)). As expected due to the greater cellular heterogeneity and genetic variation of tissue samples vs. cell lines, enhancer sets identified by different methods in the same cell line have more significant overlap than enhancer sets identified in tissues (Fig. 3b).

However, most (54%) predicted enhancers are “singletons” that are annotated by only a single enhancer identification strategy. Furthermore, the magnitude of overlap between enhancer sets is typically low: less than 50% for nearly all pairs of methods (median 17% bp overlap for K562 and 30% for liver; Fig. 3c,d; Additional file 1: Figure S2; Additional file 1: Table S2). Furthermore, the largest overlaps are in comparisons including one enhancer set with high genome coverage or in comparisons of sets that were identified based on similar data. These patterns were similar when evaluating overlap on an element-wise basis (median element-wise overlap: 18–34%; Additional file 1: Figure S3-S4, Additional file 1 : Table S2).

To further quantify overlap, we calculated the Jaccard similarity index—the number of shared bp between two enhancer sets divided by the number of bp in their union—for each pair of methods. Overall, the Jaccard similarities are also low for all contexts, with an average of 0.07 for K562 and 0.13 for liver and all pairwise comparisons below 0.35 (Fig. 3e,f, Additional file 1: Figure S2, upper triangle). Since the Jaccard similarity is sensitive to differences in set size, we also computed a “relative” Jaccard similarity by dividing the observed value by the maximum value possible given the set sizes. The relative similarities were also consistently low (Fig. 3e,f, Additional file 1: Figure S2, lower triangle).

To assess the influence of biological variation on the observed overlaps, we compared the overlap of replicates from H3K27ac ChIP-seq data in K562, Gm12878, and liver generated by the same laboratory and processed by the same peak calling pipeline (Methods). H3K27ac ChIP-seq data are used in the definition of most of the enhancer sets considered here, so high variability in this data would likely impact many of the predictions. We expected the replicates to have high overlap and serve as an “upper bound” on similarity in practical applications. On average, the replicates overlap 76% at the bp level (with a range of 54–88%) and 84% element-wise (with a range of 51–89%). The only value less than 66% comes from a single K562 comparison. Thus, while there is variation, the amount of overlap observed between enhancers identified by different methods almost always falls far below the variation between ChIP-seq replicates.

#### Enhancer sets have different levels of evolutionary conservation

**Fig. 4:**
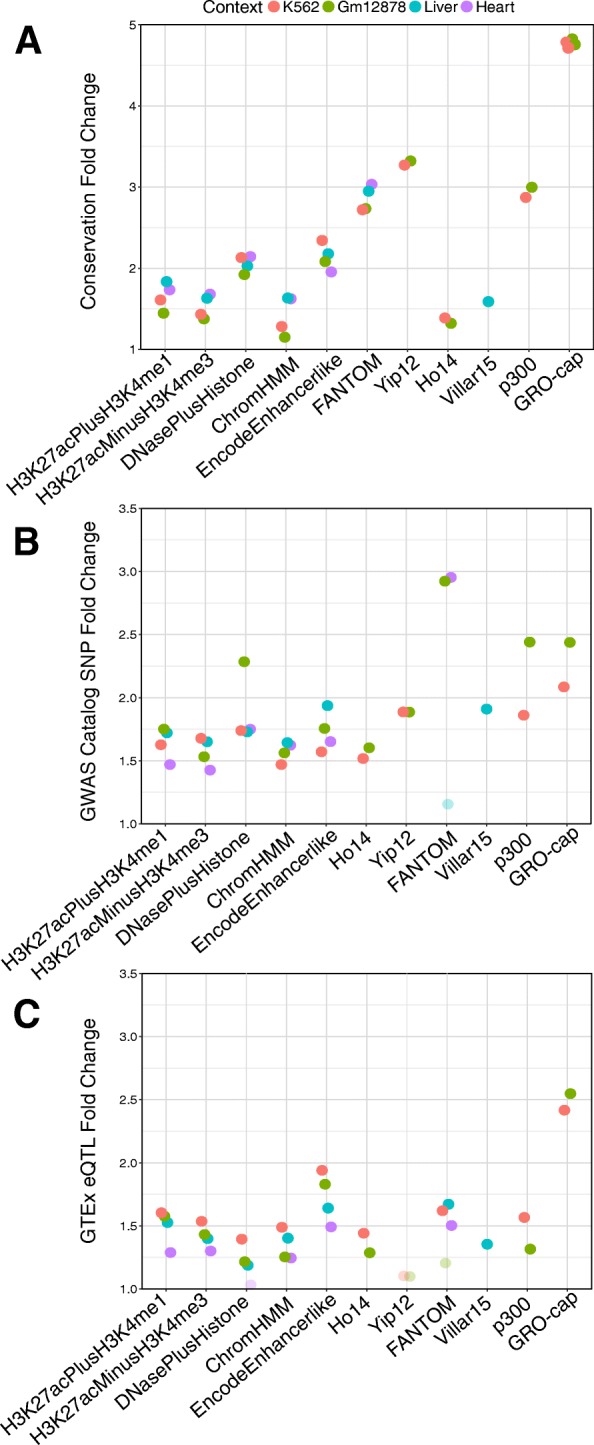
Enhancers have different levels of enrichment with functional attributes. (**a**) Enhancer sets vary in their degree of evolutionary conservation. Each point represents the enrichment (fold change compared to randomly shuffled regions) for overlap between a conserved element (combined primate and vertebrate PhastCons) and each enhancer set. Methods based on transcriptional assays and TF binding profiles (GRO-cap, FANTOM, p300, and Yip12) are the most enriched for conserved elements, while sets based on histone modification data alone are among the least enriched. (**b**) GWAS SNP enrichment among all enhancer sets for each biological context. All sets are significantly enriched, except FANTOM in K562 and liver contexts due to small sample size. (**c**) GTEx eQTL enrichment among all enhancer sets for each biological context. Transparent points indicate nonsignificant enrichment (*p* > 0.05)

Enhancers identified by different methods also differ in their levels of evolutionary constraint. Using primate and vertebrate evolutionarily conserved elements defined by PhastCons [45], we calculated the enrichment for overlap with conserved elements for each enhancer set. All enhancer sets have more regions that overlap with conserved elements than expected from length-matched regions drawn at random from the genome (Methods). However, enhancers identified by some methods are more likely to be conserved than others (Fig. 4a). Across each context, the histone-mark-based, ChromHMM, Villar15, and Ho14 enhancer sets are approximately 1.3x to 1.8x enriched for overlap with conserved elements. Adding DNaseI hypersensitivity data, as in the DNasePlusHistone and EncodeEnhancerlike sets, increases the level of enrichment slightly compared to solely histone-derived enhancers (1.9x–2.3x). In contrast, the FANTOM, Yip12, and p300 enhancers are nearly twice as enriched for conserved regions as the histone-based sets (2.7x, 3.3x, and 2.9x, respectively). GRO-cap enhancers in K562 and Gm12878 are the most enriched for overlap with conserved elements (4.7–4.8x). Evolutionary conservation was considered in the definition of the Yip12 set, but not directly in the FANTOM, p300, or GRO-cap sets. Here we considered element-wise enrichment for the number of enhancer regions overlapping conserved elements; enrichment trends are similar when we consider the number of conserved base pairs overlapped by each enhancer set (Fig. S5).

#### Identification strategies highlight different subsets of experimentally validated enhancers

Though we lack unbiased genome-wide gold-standard sets of enhancers, nearly two thousand human sequences have been tested for enhancer activity in vivo in transgenic mice at E11.5 by VISTA [46] and thousands more have been tested in cell lines via MPRAs. Strong ascertainment biases in how regions were selected for testing in these assays prevent their use as a gold standard, but they do provide an opportunity to examine overlap between validated and predicted enhancers. We evaluated the overlap and enrichment of each heart enhancer set with 1837 regions tested for enhancer activity in the developing heart by VISTA and for each annotated K562 enhancer with 15,720 regions tested in K562 cells by Sharpr-MPRA [47]. All heart enhancer sets are significantly enriched for overlap with the 126 VISTA heart positives (Fig. S6; Table S3; *p* < 0.001 for all), and each set is at least ~3x more likely to overlap validated enhancers than expected if it was randomly distributed across the genome. However, the heart enhancer sets are also significantly enriched for overlap with VISTA negatives (p < = 0.004). This is not surprising as the regions tested by VISTA were largely selected based on having evidence of enhancer activity, and they may have enhancer activity in other contexts not tested by VISTA, including adult heart. However, there is substantial disagreement among the enhancer sets about the status of the VISTA heart enhancers; 16% (*n* = 20) of validated heart enhancers are not predicted to have enhancer activity by any method, and 17% [21] are only predicted by one method (Additional file 1 :Figure S6). Similarly, all of the enhancer sets in K562 are significantly enriched for overlap with both activating and repressive regions characterized by Sharpr-MPRA (Fig. S7; *p* < 0.001 for all). There is little variation between the methods in terms of overall enrichment, with most having ~2x relative enrichment for activating regions. Nearly half of the activating regions in the MPRA (47%; 2508 / 5373) were not identified by any of the enhancer sets, and 30% of activating regions overlapping a predicted enhancer are unique to a single set (Additional file 1: Figure S7; 891 / 2747). Thus, comparisons with validated enhancers from both VISTA and MPRAs suggest that different strategies identify different subsets of active regulatory regions in the same context, and that all strategies miss a sizable portion of functional enhancer sequences. However, we again caution against interpreting the relative performance of different enhancer identification strategies on these data, since there are strong ascertainment biases in how regions were selected for testing. For example, ChromHMM enhancer predictions and DNase I hypersensitivity data were used to select the regions tested by Sharpr-MPRA.

### Interpretation of GWAS hits and eQTL is contingent on the enhancer identification strategy used

**Fig. 5:**
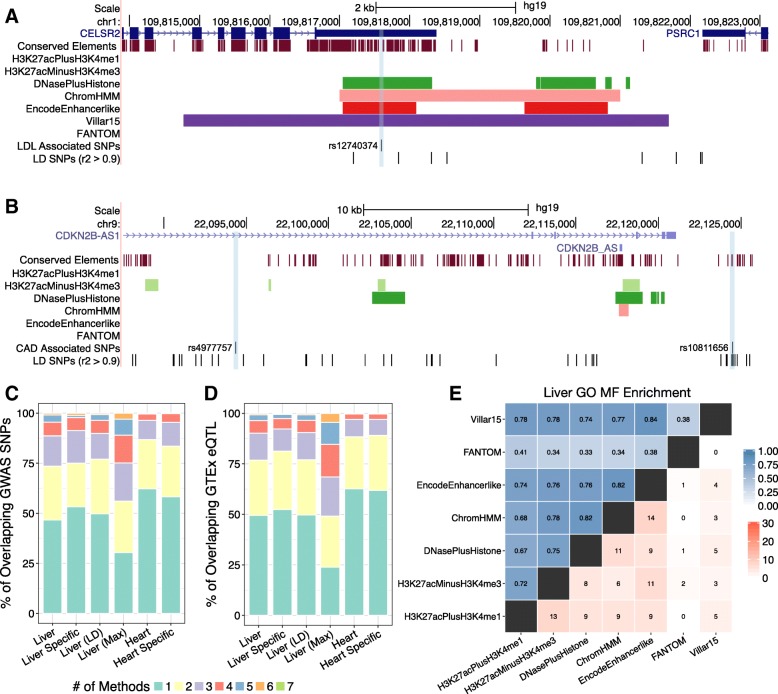
Enhancers identified by different methods differ in functional attributes. The 9 kb region on human chromosome 1 containing genetic variants associated with LDL cholesterol levels and MI in GWAS and the causal SNP (rs12740374). Here, the region containing the casual SNP is predicted to be an enhancer by four of the seven methods. GWAS tag SNPs are colored in red and LD blocks are shown with a horizontal line. (**b**) The 60 kb region of human chromosome 9 containing loci associated with coronary artery disease (CAD) in GWAS. Two of the associated variants (rs10811656 and rs4977757) have been shown to contribute to CAD risk. However, the enhancer annotations in this region are generally non-overlapping and do not highlight either functional variant. (**c**) Few GWAS SNPs overlap an enhancer; the colored bars represent the number of methods that identified the region as an enhancer. The majority of these variants are not predicted as enhancers, and very few GWAS variants are overlap enhancers from multiple methods. The conclusions are similar when considering variants in high LD (r^2^ > 0.9) with the GWAS tag SNPs in liver (Liver LD; Additional file 1: Figure S8). The pattern is also similar when limiting to SNPs associated with liver or heart related phenotypes (Liver Specific, Heart Specific). When considering the SNP in each LD block with the maximum number of enhancer overlaps there is still a large percentage of SNPs supported by none or only one method (Liver Max). This demonstrates that the situation illustrated in panel B is very common. (**d**) Among all eQTL that overlap at least one enhancer, the majority is supported by only a single method. This holds for LD- expanded and context-specific sets (Liver LD, Liver Specific, Heart Specific; Additional file 1: Figure S8). Many variants remain unique to a single method, even when limiting to the variant in each LD block overlapping the maximum of enhancer sets (Liver Max). These trends are similar to what is seen for GWAS SNPs in (**c**). (**e**) Enhancer sets from the same biological context have different functional associations. We identified Gene Ontology (GO) functional annotations enriched among genes likely to be regulated by each enhancer set using GREAT. The upper triangle represents the pairwise semantic similarity for significant molecular function (MF) GO terms associated with predicted liver enhancers. The lower triangle shows the number of shared MF GO terms in the top 30 significant hits for liver enhancer sets. Results were similar when using enhancer-gene target predictions from JEME (Additional file 1: Figure S9–10)

Functional genetic variants—in particular mutations associated with complex disease—are enriched in gene regulatory regions [5, 6]. Thus, genome-wide enhancer sets are commonly used to interpret the potential function of genetic variants observed in GWAS and sequencing studies [33, 38, 42, 48–54]. To illustrate this situation, Fig. 5a shows a 9 kb region at the human chromosome 1p13 locus containing the noncoding region between *CELSR2* and *PSRC1* associated with both low-density lipoprotein (LDL) cholesterol levels and myocardial infarction (MI) in GWAS [55]. It gives the locations of variants in high LD with the tag SNP, rs12749374, and includes regions identified as liver enhancers by the representative methods analyzed in this study. A comprehensive series of studies showed that the minor allele of rs12740374 creates a C/EBP binding site, causing increased expression of *SORT1* specifically in liver and leading to the association with both LDL cholesterol and increased risk of MI [55]. In this example, the region containing the casual SNP is predicted to be an enhancer by four of the seven methods (Fig. 5a). This represents a case in which the available enhancer data help to highlight the causal locus. As an alternative example, Fig. 5b shows a 60 kb region of human chromosome 9 that contains ten loci associated with coronary artery disease (CAD) in GWAS and other variants in high LD with the GWAS tag variants. It also shows the regions identified as enhancers by six representative methods in heart. Two of these variants (rs10811656 and rs4977757) out of 59 evaluated were recently demonstrated to disrupt binding of TEAD transcription factors in vitro and in vivo in primary human aortic smooth muscle cells, which leads to reduced expression of the cell cycle suppressor protein, p16, and contributes to CAD risk [56]. Figure 5b demonstrates that the enhancer annotations in this region are largely non-overlapping and do not highlight these two functional variants. Indeed, neither overlaps any enhancer annotation.

To explore the frequency of these scenarios genome-wide, we evaluated the overlap of GWAS loci with different enhancer identification strategies by intersecting each of the enhancer sets with 20,458 unique loci significantly associated with traits from the NHGRI-EBI GWAS Catalog. Since the GWAS catalog contains regions associated with diverse traits, we manually curated the set of GWAS SNPs into subsets associated with phenotypes relevant to liver (*n* = 346) or heart (*n* = 2127) (Table S4). We found 27% (92 / 346) of the liver associated tag SNPs and 24% (503 / 2127) of the heart associated tag SNPs overlap an enhancer predicted by at least one of strategies we considered in the appropriate context. (We consider variants in high linkage disequilibrium (LD) below.) While the amount of overlap is low, the heart and liver enhancer sets are almost universally more enriched for overlap with GWAS SNPs that influence relevant phenotypes compared to GWAS SNPs overall (Fig. 4b, Additional file 1: Table S5; 1.74x–2.68x). FANTOM enhancers are the exception to this trend due to the small number of overlapping context-specific SNPs (Additional file 1: Table S6). This suggests that the different methods, in spite of their lack of agreement, all identify regulatory regions relevant to the target context.

However, there is variation in the number of overlapping GWAS SNPs between enhancer sets, as is expected given the large variation in the number and genomic distribution of enhancers predicted by different methods (Table S6). The majority of curated GWAS liver SNPs with any enhancer overlap are overlapped by a single method (53%) and none are shared by all methods (Fig. 5c). This trend is also seen in heart, where 58% (293 / 503) of the heart associated SNPs overlapping an enhancer are identified by only a single identification strategy (Fig. 5c). This suggests that cases such as the one illustrated in Fig. 5b are far more frequent than those like Fig. 5a.

Since tag SNPs are often not the functional variants, we also considered SNPs in high LD with the GWAS SNPs (r^2^ > 0.9). The distribution of enhancer overlaps was similar when considering all candidate variants in LD (Fig. 5c), although the enrichments were lower (Fig. S8). Even after limiting to GWAS LD blocks with enhancer overlap and selecting the variant with maximum overlap between strategies, 47% (164 / 346) and 50% (1055 / 2127) are not predicted as enhancers by any method and 17% (60 / 346) and 18% (381 / 2127) are only predicted by one enhancer identification method in liver and heart, respectively (Fig. 5c). This demonstrates that enhancer maps usually disagree about which variants are likely to be functional, and that the situation illustrated in Fig. 5a is rare. Across the entire GWAS Catalog, 33% (6736 / 20,458) of SNPs overlap an enhancer predicted by at least one of the strategies in one of the contexts we considered. The trends are similar for heart, K562, and Gm12878 (Fig. 5c, Additional file 1: Figure S8). This illustrates that the annotation of variants in regions highlighted by GWAS varies greatly depending on the enhancer identification strategies used.

To test if these patterns hold for genetic variants in other functional regions, we analyzed the overlap of enhancer sets with expression quantitative trait loci (eQTL) identified by the GTEx Consortium. Enrichment for overlap with context-specific eQTL in liver or heart is generally higher than enrichment for significant eQTL overall, but the distribution of shared eQTL remains similar (Fig. 4c, Fig. 5d; Additional file 1: Table S7). Within a context, most eQTL do not overlap an enhancer, and there is wide variation in the number of eQTL overlapped by different enhancer sets (Fig. 5D; Additional file 1: Table S8). Across liver enhancer sets, 52% (2925 / 5585) of all overlapped liver eQTL and 50% (33,941 / 68,563) of general eQTL overlap an enhancer called by only one method (Fig. 5d). Considering variants in high LD (r^2^ > 0.9) does not affect this trend (Fig. 5D). After limiting the analysis to the variants with the maximum number of overlaps in each LD block, 15% (3386 / 22,234) of liver eQTL with enhancer overlap are identified by only one enhancer set (Fig. 5d). The lack of overlap is more extreme in heart, where 60% (13,925 / 22,919) heart eQTL overlap a single method, as well as K562 and Gm12878 (Fig. 5d, Additional file 1: Figure S8). Thus, in regions known to influence traits or gene regulation, the interpretation of which variants are causal varies substantially depending on the enhancer identification strategy used.

### Enhancers identified by different strategies have different functional contexts

Given the genomic dissimilarities between enhancer sets, we hypothesized that different enhancer sets from the same context would also vary in the functions of the genes they likely regulate. To test this hypothesis, we identified Gene Ontology (GO) functional annotation terms that are significantly enriched among genes likely targeted by enhancers in each set. We used two different approaches to map to genes and associated GO terms: (*i*) using the joint effect of multiple enhancers (JEME) method for mapping enhancers to putative target genes and then performing gene-based enrichment analyses, and (*ii*) applying the Genomic Regions Enrichment of Annotations Tool (GREAT) (Methods) [57, 58]. Many of the GO terms identified by both methods for the enhancer sets are relevant to the associated context (Table 2). However, most of the associated terms for the target-mapping approach were near the root of the ontologies and thus lacking in functional specificity (Table 2), likely due to the large gene target lists for most enhancer sets (Additional file 1: Table S9). As a result, we focus on the results from GREAT here, and report the results based on JEME target mapping in Additional file 1: Figure S9–S10.

**Table 2:** Top 5 Gene Ontology (Molecular Function) terms for liver enhancer sets from GREAT and JEME target-mapped WebGestalt enrichments

Enhancer Set	GO MF Terms (GREAT)	GO MF Terms (JEME+WebGestalt)
H3K27acPlusH3K4me1	cytoskeletal adaptor activity	small molecule binding
	14–3-3 protein binding	anion binding
	leukotriene-C4 synthase activity	nucleoside phosphate binding
	nucleobase-containing compound transmembrane transporter activity	nucleotide binding
	FAD binding	transferase activity
H3K27acMinusH3K4me3	14–3-3 protein binding	oxidoreductase activity
	cytoskeletal adaptor activity	anion binding
	thyroid hormone receptor binding	small molecule binding
	ARF guanyl-nucleotide exchange factor activity	nucleoside phosphate binding
	high-density lipoprotein particle binding	nucleotide binding
DNasePlusHistone	cytoskeletal adaptor activity	small molecule binding
	glucocorticoid receptor binding	anion binding
	nucleobase-containing compound transmembrane transporter activity	transferase activity
	high-density lipoprotein particle binding	nucleotide binding
	14–3-3- protein binding	nucleoside phosphate binding
ChromHMM	high-density lipoprotein particle binding	nucleotide binding
	nucleobase-containing compound transmembrane transporter activity	nucleoside binding
	cytoskeletal adaptor activity	purine nucleoside binding
	14–3-3 protein binding	DNA binding
	retinoid X receptor binding	RNA binding
EncodeEnhancerlike	cytoskeletal adaptor activity	nucleotide binding
	14–3-3 protein binding	transferase activity
	nucleobase-containing compound transmembrane transporter activity	small molecule binding
	apolipoprotein A-I binding	anion binding
	high-density lipoprotein particle binding	carbohydrate derivative binding
FANTOM	glucocorticoid receptor binding	structural constituent of ribosome
	protein kinase binding	receptor binding
	kinase binding	cell adhesion molecule binding
	methylglutaconyl-CoA hydratase activity	molecular function regulator
	vitamin D response element binding	transcription regulatory region DNA binding
Villar15	protease binding	anion binding
	phosphatidylinositol 3-kinase binding	small molecule binding
	14–3-3 protein binding	oxidoreductase activity
	cytoskeletal adaptor activity	cofactor binding
	glucocorticoid receptor binding	oxidoreductase activity, acting on CH-OH group of donors

The majority of the top 30 significant annotations from GREAT for each enhancer set are not enriched in any other set in the same context, and no terms are shared by all of the methods in a given context (Fig. 5e, lower triangle). In all of these pairwise comparisons, fewer than half of the GO terms are shared between a pair of enhancer sets. Furthermore, many of the terms shared by multiple enhancer sets are near the root of the ontology (e.g., nucleotide binding) and thus are less functionally specific. These results provide evidence that the different enhancer sets influence different functions relevant to the target biological context. These trends hold for both the Biological Process (BP) and Molecular Function (MF) ontologies and considering the top 10 and 50 annotations for each set (Figs. S11–13).

To further compare the enriched GO MF and BP annotations of each enhancer set in a way that accounts for the distance between GO terms in the ontology hierarchy and their specificity, we computed a semantic similarity measure developed for GO annotations [59, 60]. The ChromHMM and EncodeEnhancerlike enhancer sets are among the most functionally similar, with similarity scores near 0.80 in most contexts (Figs. 5e, upper triangle; Additional file 1: Figure S12). This is not surprising given that their underlying assays overlap. The functional similarity scores are lower for comparisons of the other histone modification sets, around 0.50–0.75. In all comparisons, the FANTOM enhancers have the lowest functional similarity with other enhancer sets—below 0.40 in the vast majority of comparisons in K562, liver, and heart (Fig. 5e; Additional file 1: Figure S12). FANTOM is more similar to other methods in Gm12878, with an average score of 0.59 (Additional file 1: Figure S12). As a benchmark, biological replicates of the Gm12878 H3K27ac ChIP-seq peaks received a similarity of 0.93. This suggests different functional influences for enhancer sets from the same context identified by different methods, with FANTOM as a particular outlier. We note that enhancer target gene identification remains a challenging problem, and both strategies for mapping enhancers to potential target genes considered here (GREAT and JEME) likely include false positives and negatives. However, insofar as they reflect the genomic context of the different enhancer sets, they reveal significant functional differences between enhancer identification methods.

### Genomic and functional clustering of enhancer sets

Our analyses of enhancer sets within the same biological context reveal widespread dissimilarity in both genomic and functional features. To summarize and compare the overall genomic and functional similarity of the enhancer sets across contexts, we clustered them using hierarchical clustering and MDS based on their Jaccard similarity in genomic space and the GO term functional similarity of predicted target genes (Methods).

**Fig. 6:**
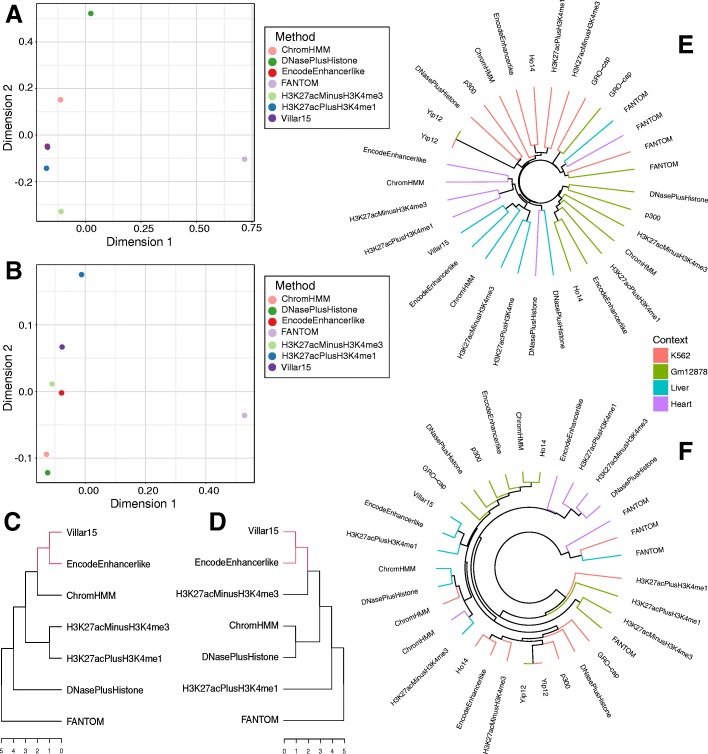
The genomic and functional similarities between enhancer sets are not consistent. (**a**) Multidimensional scaling (MDS) plot of liver enhancer sets based on the Jaccard similarity of the genomic distributions (Fig. 3b). (**b**) MDS plot for liver enhancers based on distances calculated from molecular function (MF) Gene Ontology (GO) term semantic similarity values with GREAT (Fig. 5**e**). (**c**, **d**) Ranked hierarchical clustering based on the Jaccard similarities of the genomic distributions (**c**) of all liver enhancer sets compared to clustering based on GO semantic similarity (**d**). FANTOM enhancers are the most distant from all other enhancer sets in both genomic and functional similarity, but the relationships between other sets are not conserved. Red branches denote identical subtrees within the hierarchy. (**e**) Hierarchical clustering based on genomic Jaccard distances for all contexts and methods with annotations in each context. (**f**) Hierarchical clustering of all available enhancer sets based on GO term distances. Terminal branches are colored by biological context. With the exception of FANTOM enhancers, the enhancer sets’ genomic distributions are more similar within than between biological contexts. Functional similarity does not always correlate with genomic similarity, and the clustering by biological context is weaker in functional space

Several trends emerged from analyzing the genomic and functional distribution within and between biological contexts. First, the FANTOM eRNA and GRO-cap enhancers are consistently distinct from all other enhancer sets in both their genomic distribution and functional associations (Fig. 6). Differences between eRNA and non-eRNA enhancer sets appear to dominate any other variation introduced by biological, technical, or methodological differences. This is also true when comparing patterns of TF binding site motif enrichment in different enhancer sets. FANTOM and GRO-cap are consistent outliers with the largest differences in predicted TF binding site enrichment from the other methods (Fig. S14; Additional file 2: Table S10).

A second trend in these comparisons is that similarity in genomic distribution of enhancer sets does not necessarily translate to similarity in functional space, and vice versa. For example, although EncodeEnhancerlike regions are close to ChromHMM and the histone-derived H3K27acPlusH3K4me1 set and the machine learning models in the genomic-location-based projection (Fig. 6a,c), they are located far from those sets in the functional comparisons and hierarchical clustering (Fig. 6b,d).

Finally, comparing enhancer sets by performing hierarchical clustering within and between biological contexts reveals that genomic distributions are generally more similar within biological contexts, compared to other sets defined by the same method in a different context (Fig. 6e). For example, the ChromHMM set from heart is more similar to other heart enhancer sets than to ChromHMM sets from other contexts. In contrast, the enhancer set similarities in functional space are less conserved by biological context (Fig. 6f). Here, the heart ChromHMM set is functionally more similar to the H3K27acMinusH3K4me3 set from liver cells than other heart enhancer sets. In general, cell line enhancer sets (red and green) show more functional continuity than heart and liver sets (blue and purple). However, FANTOM enhancers are the exception to these trends; FANTOM enhancers from each context form their own cluster based on their genomic distribution, underscoring their uniqueness. GRO-cap enhancers cluster with FANTOM in the genomic location clustering and with their cellular contexts in the functional clusters.

### Combining enhancer sets does not strongly increase evidence for regulatory function

**Fig. 7:**
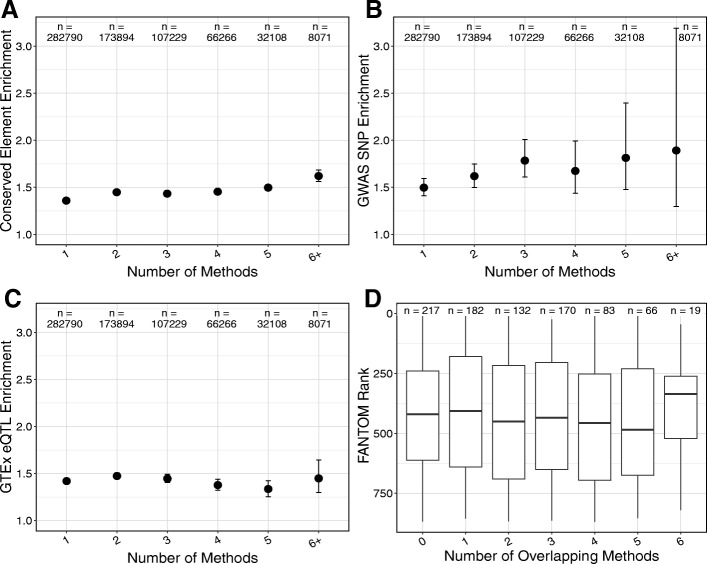
Enhancers identified by multiple methods have little additional evidence of function. (**a**) Enrichment for overlap between conserved elements (*n* = 3,930,677) and liver enhancers stratified by the number of identification methods that predicted each enhancer. (**b**) Enrichment for overlap between GWAS SNPs (*n* = 20,458) and liver enhancers stratified by the number of identification methods that predicted each enhancer. (**c**) Enrichment for overlap between GTEx eQTL (*n* = 429,964) and liver enhancers stratified by the number of identification methods that predicted each enhancer. In (**a**-**c**), the average enrichment compared to 1000 random sets is plotted as a circle; error bars represent 95% confidence intervals; and n gives the number of enhancers in each bin. The only significant differences are found in the enrichment for evolutionary conservation (**a**), but the difference is modest in magnitude (1.36x for 1 vs. 1.62x for 6+). (**d**) Boxplots showing the distribution of confidence score ranks for FANTOM enhancers in liver partitioned into bins based on the number of other methods that also identify the region as an enhancer. Lower rank indicates higher confidence; note that the y-axis is flipped so the high confidence (low rank) regions are at the top. The lack of increase in enhancer score with the number of methods supporting it held across all methods tested (Additional file 1: Figure S14–17)

Although there are large discrepancies in genomic and functional attributes between enhancer sets identified by different methods in the same context, we hypothesized that the subset of regions shared by two or more sets would have stronger enrichment for markers of gene regulatory function. To test this, we analyzed whether regions identified by multiple methods have increased “functional support” compared to regions identified by fewer methods. We evaluated three signals of functional importance: *(i)* enrichment for overlap with evolutionarily conserved elements, *(ii)* enrichment for overlap with GWAS SNPs, and *(iii)* enrichment for overlap with GTEx eQTL. For each, there are only small changes as the number of methods identifying a region increases (Fig. 7a-c). Regions identified as enhancers by more than one method are slightly more enriched for conserved elements compared to the genomic background, but there is little difference among regions identified by 2–5 methods (Fig. 7a). Regions predicted by 6 or more methods are significantly more enriched for conserved elements than those with less support, but effect size is modest (1.36x for 1 vs. 1.62x for 6+). There is a modest increase in the enrichment for overlap with GWAS SNPs among enhancers identified by more identification methods (1.50x for 1 vs. 1.89x for 6+); however, given the relatively small number of GWAS SNP overlaps, none of these differences were statistically significant (Fig. 7b). We observed no increase in the enrichment for overlap with eQTL as the support for enhancer activity increased (Fig. 7c). Thus, we do not find strong evidence of increased functional importance in enhancers identified by multiple methods compared to enhancers identified by a single method. Importantly, this implies that intersecting enhancer identification strategies will focus on a smaller set of enhancers with only modest evidence for increased functional relevance.

Several enhancer identification methods provide confidence scores that reflect the strength of evidence for each enhancer. We hypothesized that high confidence enhancers from one method would be more likely to overlap enhancers identified by other methods. To test this, we ranked each enhancer based on its confidence or signal, with a rank of 1 representing the highest confidence in the set. There was no clear trend between the confidence score of an enhancer from one method and the number of methods that identified the region as an enhancer (Fig. 7d, Additional file 1: Figure S15–S18). Overall, enhancers identified by multiple methods show a similar confidence distribution when compared to regions identified by a single method. Indeed, for some enhancer sets the median score decreases as the regions become more highly shared (Additional file 1: Figure S15, S18). This provides further evidence that building enhancer sets by simple combinations of existing methods is unlikely to lead to a higher confidence subset, and that filtering based on simple agreement between methods may not substantially improve the specificity of enhancer predictions.

Additionally, we do not find evidence that analyzing only the top enhancer predictions from each method changes the results reported here. Using the five enhancer identification strategies with confidence or signal rankings in each context we compared the top 100, 500, and 800 enhancer predictions. There remains little sharing between these subsets, with most enhancer regions remaining unique to a single method. Furthermore, the level of enrichment for overlap with functional attributes remains largely similar, but less significant in many cases (Additional file 1: Figure S19-S21).

### A tool for evaluating of the robustness of conclusions across enhancer identification strategies

Our findings imply that results of studies of enhancers are sensitive to the enhancer identification strategy used. To facilitate evaluation of the sensitivity and robustness of results with respect to this choice, we provide an integrated database (creDB) of predicted enhancer sets. It contains all putative enhancer sets used in this study, and enhancer sets from multiple primary and meta-analyses annotating enhancers across cellular contexts. In contrast to other efforts providing large compilations of enhancer sets [50, 51], creDB is not a web-server or “enhancer browser”. Rather, it is an SQL database with a convenient interface to the R programming language and distributed as an R package. creDB is designed to enable users to easily consider many different genome-wide sets of predicted enhancers in their bioinformatics analyses and to evaluate the robustness of their findings. The current version of creDB contains more than 3.5 million predicted enhancers and is available online (Methods).

## Discussion

Accurate enhancer identification is a challenging problem, and recent efforts have produced a variety of experimental and computational approaches. Each method, either explicitly or implicitly, represents a different perspective on what constitutes an enhancer and which identifiable signatures are most informative about enhancer activity. For example, histone modifications characteristic of enhancers are found on histones that flank active enhancers, while eRNA are thought to be bidirectionally transcribed from the active sequence itself. As a result, despite the use the term “enhancer” to describe all these regions in the literature, we expected different assays and algorithms to identify somewhat different sets of regions. Given the lack of comprehensive genome-wide “gold standard” enhancer sets, evaluation of the accuracy of these approaches is challenging. Thus, we compared existing strategies with respect to one another and to proxies for regulatory function. All pairs of enhancer sets overlap more than expected by chance, but we found substantial differences in the genomic, evolutionary, and functional characteristics of identified enhancers *within* similar tissues and cell types. Enhancer sets vary significantly in their overlap with conserved genomic elements, GWAS loci, and eQTL. Furthermore, the majority of GWAS loci and eQTL have inconsistent evidence of enhancer function across enhancer sets. In addition, regions identified as enhancers by multiple methods *do not* have significantly stronger evidence of regulatory function.

The consistent lack of agreement between methods demonstrates that many working definitions of “enhancer” have very low overlap. Focusing on functional annotations, we find agreement between methods about basic functions, but substantial differences in more specific annotations. This suggests that different strategies contribute unique information towards the identification of functionally important enhancers. In general, enhancers defined by FANTOM have modestly more enrichment for proxies of functional activity than other methods, but this comes at the expense of low sensitivity (e.g., Figs. S6–7). Our results argue that, given the lack of a clear gold standard and the substantial disagreement between strategies, it does not make sense to identify a single “best” method given current knowledge.

Furthermore, because enhancer identification strategies have such substantial differences, one strategy cannot and should not be used as a proxy for another. Using different strategies can yield substantially different biological interpretations and conclusions, e.g., about the gene regulatory potential of a genetic variant or the degree of evolutionary constraint on enhancers. This is particularly important given that studies of gene regulation commonly use only a single approach to define enhancers. GWAS have identified thousands of non-coding loci associated with risk for complex disease, and a common first step in the interpretation of a trait-associated locus is to view it in the context of genome-wide maps of regulatory enhancer function [33, 38, 42, 52–54, 61, 62]. Thus, our findings complicate the use of annotated enhancers to study the mechanisms of gene regulation and to elucidate the molecular underpinnings of disease, most notably in non-coding variant prioritization [30, 63, 64].

Technical and biological variation in the underlying experimental assays or data processing pipelines likely contribute to the variation between putative enhancer sets. However, we minimized technical variation by calling and comparing enhancers using consistent computational pipelines. Furthermore, comparisons of biological replicates of histone modification ChIP-seq data suggest that the level of difference we observe between enhancer sets is larger than among biological replicates. Genetic variation between individuals could also explain some of the discordance. Previous work shows that chromatin states associated with weak enhancer activity exhibit some variation between individuals, and QTL associated with changes in epigenetic modifications and enhancer activity between individuals have been identified [65, 66]. However, the proportion of epigenetic modifications that are variable across individuals is estimated to be small (1–15%) [67]. Thus, variation between individuals is unlikely to be the main cause of the lack of agreement we observe between methods, in particular for enhancer sets defined from cell lines. Furthermore, there are strong similarities between enhancers and other regulatory elements, like promoters, and some promoters even have enhancer activity [2, 68, 69]. We focus on methods designed to distinguish enhancers to reduce the impact of disagreement due to the comparison of different elements from the broader regulatory spectrum; nevertheless, some identification strategies may include or exclude functional elements with variable activities. However, while the proxies we use for regulatory function (evolutionary conservation, GWAS loci, and eQTL) each have weaknesses, we observe similar disagreement across each proxy. This supports the functional relevance of the differences we demonstrate between enhancer sets. Taking each of these limitations into account, the disagreements we observe remain striking.

## Conclusions

In light of the complexity of enhancer identification, what should we do? First, we must resist the convenience of ignoring it. When interpreting non-coding variants of interest or characterizing the enhancer landscape in a new biological context, we must be mindful that using a single identification strategy is insufficient to comprehensively catalog enhancers. Different assays and algorithms have different attributes, and we suggest employing a range of approaches to obtain a more robust view of the regulatory landscape. To facilitate this, we have developed creDB, a comprehensive, easily queried database of over 3.5 million putative enhancer annotations. However, simply focusing on variants with multiple lines of evidence of enhancer activity will not solve the problem, especially when our ability to quantify the false positive rate in a genome-wide enhancer map is limited. More sophisticated statistical models of enhancers and their properties are needed in order to interpret non-coding variants of interest. Previous work has shown that integrating diverse genomic, evolutionary, and functional data can improve the ability to distinguish validated enhancers from the genomic background [29], but obtaining a concordant and functionally relevant set of enhancers remains challenging. We are hopeful that new experimental techniques, like MPRAs, and biologically motivated machine learning methods for integrating different definitions of enhancers will yield more consistent and specific annotations of regions with regulatory functions.

Second, our study highlights the need for more refined models of the architecture and dynamics of *cis*-regulatory regions. Many different classes of regions with enhancer-like regulatory activities have been discovered [4, 14, 19, 30, 34, 70, 71]. We argue that collapsing the diversity of vertebrate distal gene regulatory regions into a single category is overly restrictive. Simply calling all of the regions identified by these diverse approaches “enhancers” obscures functionally relevant complexity and creates false dichotomies. While there is appreciation of this subtlety within the functional genomics community, there is still a need for more precise terminology and improved statistical and functional models of the diversity of *cis*-regulatory “enhancer-like” sequences and their architectures. Given this diversity, we should not expect all results to be robust to the enhancer identification strategy used.

Finally, we believe that ignoring enhancer diversity impedes research progress and replication, since “what we talk about when we talk about enhancers” includes diverse sequence elements across an incompletely understood spectrum, all of which are likely important for proper gene expression. Efforts to stratify enhancers into different classes, such as poised and latent, are steps in the right direction, but are likely too coarse given our incomplete current knowledge. We suspect that a more flexible model of distal regulatory regions is appropriate, with some displaying promoter-like sequence architectures and modifications and others with distinct regulatory properties in multiple, potentially uncharacterized, dimensions [2, 72, 73]. Consistent and specific definitions of the spectrum of regulatory activity and architecture are necessary for further progress in enhancer identification, successful replication, and accurate genome annotation. In the interim, we must remember that genome-wide enhancer sets generated by current approaches should be treated as what they are—incomplete snapshots of a dynamic process.

## Methods

In this section, we first describe the different enhancer identification strategies that we consider. We then describe how we obtained various annotations of functionally relevant attributes for these enhancers. Finally, we describe the analytical approaches used to compare the enhancer sets to one another in terms of their genomic locations and annotations.

### Enhancer identification methods

Here, we summarize how we defined human enhancer sets across four biological contexts. All analyses were performed in the context of the GRCh37/hg19 build of the human genome. We used TSS definitions from Ensembl v75 (GRCh37.p13).

We downloaded broad peak ChIP-seq data for three histone modifications, H3K27ac, H3K4me1, and H3K4me3 from the ENCODE project [32] for two cell lines, K562 and Gm12878, and from the Roadmap Epigenomics Consortium [74] for two primary tissues, liver and heart. The ENCODE broad peaks were generated by pooling data from two isogenic replicates. The Roadmap Epigenomics broad peaks were also generated with data from two replicates. We chose broad peak files because we expect histone modifications to flank active enhancer regions, and the broad peaks represent wide regions of relative enrichment that are likely to encompass the functionally relevant sequences. See below for details on consistent peak calling. We downloaded the “enhancer-like” annotations from ENCODE (version 3.0); these combine DHSs and H3K27ac ChIP-seq peaks using an unsupervised machine learning model. We retrieved ChromHMM enhancer predictions [75] for the K562 and Gm12878 cell lines from the models trained on ENCODE data [15]. We downloaded ChromHMM predictions for liver and heart tissues from the 15-state segmentation performed by the Roadmap Epigenomics Consortium. For all ChromHMM sets, we combined the weak and strong enhancer states. We considered two enhancer sets for K562 and Gm12878 based on supervised machine learning techniques—one described in Yip et al. 2012 [43], and the other in Ho et al. 2014 [44]. Yip12 predicted ‘binding active regions’ (BARs) using DNA accessibility and histone modification data; the positive set contained BARs overlapping a ‘transcription-related factor’ (TRF), and the negative set contained BARs with no TRF peaks. The predicted regions were filtered using other genomic characteristics to determine the final set of enhancers [43]. Ho14 used H3K4me1 and H3K4me3 ChIP-seq peaks in conjunction with DHSs and p300 binding sites to predict regions with regulatory activity both distal and proximal to TSSs. The distal regulatory elements make up their published enhancer set [44]. For K562 and Gm12878 we also downloaded p300 ChIP-seq peaks from ENCODE [32]. We downloaded enhancer regions predicted by the FANTOM consortium for the four sample types analyzed [25]. We also downloaded enhancer predictions in liver from Villar et al. 2015 [35]. We downloaded regions of nascent bidirectional transcription from GRO-cap data generated for the two cell lines, K562 and Gm12878 [39]. The transcribed regions on matched positive and negative strands were merged into a single annotation.

To represent enhancer identification strategies in common use, we created two additional enhancer sets for this study using histone modification ChIP-seq peaks and DNase-seq peaks downloaded from ENCODE and Roadmap Epigenomics. The H3K27acPlusH3K4me1 track is a combination of H3K27ac and H3K4me1 ChIP-seq peak files [4, 19, 34]. If both types of peaks were present (i.e., the regions overlap by at least 50% of the length of one of the regions) the intersection was classified as an enhancer. Similarly, to create the H3K27acMinusH3K4me3 set for each context, we intersected H3K27ac and H3K4me3 ChIP-seq peak files and kept regions where H3K27ac regions did not overlap a H3K4me3 peak by at least 50% of their length. We derived the combination of H3K27ac and H3K4me3 and the 50% overlap criterion from previous studies [14, 34, 35]. The DNasePlusHistone track is based on the pipeline described in Hay et al. 2016 [36]. It combines H3K4me1, H3K4me3, DNaseI hypersensitive sites (DHSs), and transcription start site (TSS) locations. We filtered a set of DHSs, as defined by DNase-seq, for regions with an H3K4me3 / H3K4me1 ratio less than 1, removed regions within 250 bp of a TSS, and called the remaining regions enhancers.

For all enhancer sets, we excluded elements overlapping ENCODE blacklist regions and gaps in the genome assembly [76]. Additionally, due to the presence of extremely long regions in some enhancer sets, likely caused by technical artifacts, we removed any regions more than three standard deviations above or below the mean length of the dataset. This filtering process removed relatively few annotations (Table S11).

When considering the agreement between biological replicates for K562, Gm12878, and liver H3K27ac ChIP-seq data, we downloaded the FASTQ files from ENCODE [32] and Villar et al. 2015 [35], respectively, aligning each to GRCh37.p13 using BWA [77] (v.0.7.15, default options). We called peaks of broad enrichment using MACS [78] (v.1.4.2, default options). We processed each of the replicate peak files using the same pipeline as the published peak files.

### Enhancer attribute data

We downloaded evolutionarily conserved regions defined by PhastCons, a two-state hidden Markov model that defines conserved elements from multiple sequence alignments [45]. We concatenated primate and vertebrate PhastCons elements defined over the UCSC alignment of 45 vertebrates with humans into a single set of conserved genomic regions. We downloaded the full list of 20,458 unique GWAS SNPs from the GWAS Catalog (v1.0, downloaded 08-10-2016) [79]. We also manually curated the set of GWAS SNPs into subsets associated with phenotypes relevant to liver or heart for context-specific analyses (Table S4). We downloaded all GTEx eQTL from the GTEx Portal (v6p, downloaded 09-07-2016) [80]. We concatenated the data from all 44 represented tissues and ran the enrichment analysis on unique eQTL, filtering at four increasingly strict significance thresholds: 10^− 6^, 10^− 10^, 10^− 20^, and 10^− 35^. We present the results from the *p*-value threshold of 10^− 10^, although the choice of threshold did not qualitatively alter the results. We also performed separate context-specific analyses on liver and heart specific eQTL from GTEx (*p* < 10^− 10^). To identify other variants tagged by the GWAS SNPs and eQTL, we expanded each set to include SNPs in high LD (r^2^ > 0.9) in individuals of European ancestry from the 1000 Genomes Project (phase 3) [81].

Experimentally validated enhancer sequences with activity in the heart and all negative enhancer sequences were downloaded from the VISTA enhancer browser (downloaded 11-16-2017) [46]. We also downloaded sequences and Sharpr-MPRA activity levels for 15,720 putative enhancer regions tested for regulatory activity in K562 cells using a massively parallel reporter assay (MPRA) [47]. The Sharpr-MPRA algorithm infers a regulatory score for each base pair in a region using a probabilistic model, with positive scores indicating activating regulatory regions and negative scores indicating repressive regions. Following Ernst et al., we summarized the overall regulatory activity of a given enhancer region as the activity value with the maximum absolute value and classified the enhancer regions into activating (*n* = 5373) and repressive (*n* = 10,347) based on the score’s sign [47].

### Genomic region overlap and similarity

To quantify genomic similarity, we calculated the base pair overlap between two sets of genomic regions, *A* and *B*, by dividing the number of overlapping base pairs in *A* and *B* by the total number of base pairs in *B*. We also performed this calculation on element-wise level, by counting the number of genomic regions in *B* overlapping regions in *A* by at least 1 bp, and dividing by the number of genomic regions in *B*. We performed both calculations for each pairwise combination of enhancer sets. All overlaps were computed using programs from the BEDtools v2.23.0 suite [82].

We also evaluated the similarity between pairs of genomic region sets using the Jaccard similarity index. The Jaccard index is defined as the cardinality of the intersection of two sets divided by cardinality of the union. In our analyses, we calculated the Jaccard index at the base pair level. We also computed the relative Jaccard similarity as the observed Jaccard similarity divided by the maximum possible Jaccard similarity for the given sets of genomic regions, i.e., the number of bases in the smaller set divided by the number of bases in the union of the two sets. To visualize overlaps, we plotted heatmaps for pairs of methods using ggplot2 in R [83].

### Genomic region overlap enrichment analysis

To evaluate whether the observed base pair overlap between pairs of enhancer sets is significantly greater than would be expected by chance, we used a permutation-based approach. We calculated an empirical *p*-value for an observed amount of overlap based on the distribution of overlaps expected under a null model of random placement of length-matched regions throughout the genome. We used the following protocol: let *A* and *B* denote two sets of genomic regions; count the number of bp in *A* that overlap *B*; generate 1000 random sets of regions that maintain the length distribution of *B*, excluding ENCODE blacklist regions and assembly gaps; count the number of bp in *A* that overlap regions in each of the random sets; compare the observed bp overlap count with the overlap counts from each iteration of the simulation and compute a two-sided empirical *p*-value. We used the same framework to evaluate element-wise comparisons by counting the number of regions in *A* that overlap *B* rather than the bps. This approach was performed using custom Python scripts and the Genomic Association Tester (GAT) [84]. We note that this measure of overlap significance is not symmetric, and accordingly we confirmed results of our element-wise results for both orderings of the pairs of enhancer sets.

### Enhancer conservation, GWAS catalog SNP, and GTEx eQTL enrichment

In addition to comparing the overlap between pairs of enhancer sets, we also computed enrichment for overlap of evolutionarily conserved regions, GWAS SNPs, and GTEx eQTL with each of the enhancer sets. For conserved elements, we proceeded as described above for comparisons between pairs of enhancer sets, but considered the conserved elements as set *A* and the enhancers as set *B*. For GWAS tag SNPs, we considered each variant as a region in set *A* and the enhancer regions as set *B*. We used the same approach for testing all variants in LD (r^2^ > 0.9) with GWAS tag SNPs and for testing enrichment for liver- and heart-specific GWAS tag SNP sets. We also tested for enrichment using only the variant with the maximum number of enhancer set overlaps for each GWAS SNP’s LD block. In this analysis, *A* was the set of variants with maximum enhancer set overlap for each LD block and *B* was the set of enhancers. Enrichments were computed for the eQTL SNP sets using the same strategy as described for GWAS SNPs.

### Enhancer set gene ontology annotation and similarity

We used GREAT to find Gene Ontology (GO) annotations enriched among genes nearby the enhancer sets. GREAT assigns each input region to regulatory domains of genes and uses both a binomial and a hypergeometric test to discover significant associations between regions and associated genes’ GO annotation terms [57]. Due to the large number of reported regions in each enhancer set, we considered significance based only on the binomial test with the Bonferroni multiple testing correction (< 0.05). We downloaded up to 1000 significant terms for each enhancer set from the Molecular Function (MF) and Biological Process (BP) GO ontologies. We calculated the similarity between lists of GO terms using the GOSemSim package in R [60]. GOSemSim uses sematic similarity metric that accounts for the hierarchical organization and relatedness of GO terms when calculating the similarity score [59]. For each pair of enhancer sets, we calculated the similarity between their associated GO terms, and converted the resulting similarity matrix into a dissimilarity matrix. We also calculated the number of shared GO terms between pairs of methods and manually compared the top ten significant terms for each enhancer set.

Since enhancers often target genes over long distances, we also considered target predictions generated by the JEME algorithm to assign enhancers to potential target genes in each context [58]. JEME is a two-step process that considers the superset of all enhancers across contexts as well as context-specific biomarkers to make its predictions. By intersecting each enhancer set with the corresponding enhancer-target maps from JEME, we created a set of putatively regulated genes for each method in a given context. We performed GO enrichment analyses on the gene sets using the online tool WebGestalt [85]. We downloaded the top 1000 significant terms (*p* < 0.05 after Bonferroni correction) for each enhancer set from the BP and MF GO ontologies and calculated the pairwise similarity between lists of GO terms using the same semantic similarity metric as above.

### Enrichment for known transcription factor motifs

We used AME from the MEME suite to quantify enrichment for known motifs from the HOCOMOCO (v11) core database in each enhancer set [86, 87]. Enrichment was calculated based on a comparison to background sequences generated by randomly shuffling the enhancer sequences while maintaining their dinucleotide frequency distribution. We used the default *E*-value threshold of 10 to define significant enrichment. We calculated the similarity between sets of enriched motifs using the Jaccard index.

### Genomic and functional clustering of enhancer sets

To identify groups of similar enhancers in genomic and functional space, we performed hierarchical clustering on the enhancer sets. For genomic similarity, we converted the pairwise Jaccard similarity to a dissimilarity score by subtracting it from 1 and then clustered the enhancer sets based on these values. For functional similarity, we clustered the lists of GO terms returned by GREAT for each enhancer set or sets of enriched TF binding motifs from AME. We calculated similarity of GO terms using the GoSemSim package in R and converted it to dissimilarity by subtracting the similarity score from 1. For both the genomic and GO similarity, we used agglomerative hierarchical clustering in R function with the default complete linkage method to iteratively combine clusters [88]. We visualized the cluster results as dendrograms using ggplot2 and dendextend [83, 89]. We performed multidimensional scaling (MDS) on the Jaccard, GO term, and TF motif dissimilarity matrices using default options in R [88].

### Combinatorial analysis of enhancer sets and enrichment for functional signals

We stratified genomic regions by the number of enhancer identification strategies that annotate them in order to determine whether regions predicted to be enhancers by more methods show greater enrichment for three signals of function—evolutionarily conserved base pairs, GWAS SNPs, or GTEx eQTL—compared to regions with less support. We divided all regions predicted by any enhancer identification method in a given context into bins based on the number of methods that predicted it. Some enhancer regions had varying prediction coverage and were split across multiple bins. While infrequent (< 3% of regions), we removed all regions less than 10 bp in length since these are unlikely to function as independent enhancers. For each enhancer support bin, from 1 to the number of prediction methods, we calculated the enrichment for overlap with each functional signal using the permutation framework described above. We considered three different proxy sets: evolutionarily conserved base pairs as defined by PhastCons elements, GWAS SNPs, and GTEx eQTL. In each enrichment analysis, the functional signal regions were set *A* and the enhancer regions with a given level of support were set *B*. We report the average enrichment for each enhancer support bin with the empirical 95% confidence intervals.

For enhancer sets with quantitative enhancer-level scores available across contexts, we ranked each enhancer by its score, and then analyzed whether regions that have higher scores are more likely to be predicted by other identification methods. We calculated the rank using the ChIP-seq or CAGE-seq signal scores for a subset of methods (H3K27acPlusH3K4me1, H3K27acMinusH3K4me3, DNasePlusHistone, FANTOM), and the machine learning derived score for EncodeEnhancerlike regions. Within each set, we sorted the enhancer regions by score and assigned ranks starting at 1 for the top-scoring region. We then partitioned the enhancer regions in each set by the number of other enhancer sets that overlap at least one base pair in that region. To compare the most confident enhancer predictions, we subset each of the ranked methods into the top 100, 500, or 800 top enhancers. We used these subsets to calculate the level of enrichment for overlap with GWAS Catalog SNPs, GTEx eQTL, and evolutionary conservation based on the number of methods that agree on each annotated region.

### Enhancer database

We created creDB, a database of predicted *cis*-regulatory elements. It currently contains all 1,371,867 predicted enhancers analyzed in this study and representative sets from other common contexts. It is implemented as an SQLite database with an R interface and distributed as an R package. Our design facilitates the integration of creDB into a wide range of genome-wide bioinformatics workflows, alleviating the vetting and processing that is necessary with flat file downloads. This sets it apart from other efforts that collect enhancers but focus on providing interfaces for searching a small number of candidates or for “browsing” enhancers on a case-by-case basis. For all enhancers, creDB contains information about genomic location, identification strategy, and tissue or cell type of activity. We envision creDB as a growing community resource and encourage other researchers to contribute.

## Additional files

Additional file 1: **Figure S1.** Enhancer sets across all contexts considered differ in both number (A) and length (B): Gm12878; Heart. **Figure S2.** Enhancer sets have low amounts of overlap with each other in bp-wise comparisons. **Figure S3.** Enhancer sets overlap more than expected by chance in element-wise comparisons. **Figure S4.** Enhancer sets have low amounts of overlap with each other in element-wise comparisons. **Figure S5.** Enhancers identified by different methods differ in enrichment for base pair overlap with functional attributes. **Figure S6.** Enhancer identification strategies recognize different subsets of validated enhancers. **Figure S7.** K562 enhancer sets have similar low levels of enrichment for activating regions validated by Sharpr-MPRA. **Figure S8.** Even among the variants in each functional LD block (r^2^ > 0.9) with the most enhancer set overlap, there is substantial disagreement between enhancer identification methods. **Figure S9.** Pairwise similarity for GO Molecular Function (MF) enrichments for enhancer sets based on JEME’s putative mappings to target genes in K562 (A), Gm12878 (B), liver (C), and heart (D). **Figure S10.** Pairwise similarity for GO Biological Process (BP) for enhancer sets based on JEME’s putative mappings to target genes in K562 (A), Gm12878 (B), liver (C), and heart (D). **Figure S11.** Pairwise similarity for GO Molecular Function (MF) enrichments from GREAT for liver enhancer sets. **Figure S12.** There is low pairwise similarity between GO Molecular Function (MF) enrichments calculated with GREAT for enhancer sets in the same context. **Figure S13.** There is low pairwise similarity between GO Biological Process (BP) enrichments calculated with GREAT for enhancer sets in the same context. **Figure S14.** Clustering enhancer sets on similarity of enriched transcription factor binding motifs illustrates different clustering of methods. **Figure S15.** Regions identified as enhancers by multiple methods do not have higher confidence scores than regions identified by a single method. **Figure S16.** Score distributions for K562 enhancer sets are similar between regions identified as enhancers by a single method and those identified by multiple methods: (A) H3K27acPlusH3K4me1, (B) H3K27acMinusH3K4me3, (C) DNasePlusHistone, (D) EncodeEnhancerlike, and (E) FANTOM. **Figure S17.** Score distributions for Gm12878 enhancer sets are similar between regions identified as enhancers by a single method and those identified by multiple methods: (A) H3K27acPlusH3K4me1, (B) H3K27acMinusH3K4me3, (C) DNasePlusHistone, (D) EncodeEnhancerlike, and (E) FANTOM. **Figure S18.** Score distributions for heart enhancer sets are similar between regions identified as enhancers by a single method and those identified by multiple methods: (A) H3K27acPlusH3K4me1, (B) H3K27acMinusH3K4me3, (C) DNasePlusHistone, (D) EncodeEnhancerlike, and (E) FANTOM. **Figure S19.** Enrichment for functional attributes is not significantly different between regions identified as enhancers by a single method and those identified by multiple methods when focusing on the top 100 predictions from each method. **Figure S20.** Same as Fig. S19, but considering the top 500 predictions from each method. **Table S1.** The average distance (in bp) to the closest TSS over all enhancers identified by each method in each cellular context. **Table S2.** Summary statistics for pairwise percent overlap, both in a base pair and element-wise comparison. **Table S3.** Number of observed VISTA heart positive and VISTA negative overlaps for each context and enhancer identification method. **Table S4.** Curated list of relevant GWAS phenotypes for liver (*n* = 50) and heart (*n* = 169). **Table S5.** Enrichments for overlap with context-specific SNPs in liver and heart. **Table S6.** Number of overlapping GWAS SNPs per enhancer identification method and context. **Table S7.** Enrichments for overlap with context-specific eQTL in liver and heart. **Table S8.** Number of overlapping GTEx eQTL per enhancer identification method and context. **Table S9.** Number of target genes mapped to each enhancer set by JEME. For K562 and Gm12878, p300 and GRO-cap are not included in this mapping. **Table S11.** Number of enhancers removed by length filtering. (DOCX 36740 kb)

Additional file 2: **Table S10.** Motif IDs and *E*-values for significantly enriched transcription factor binding motifs from the HOCOMOCO (v11) core database for enhancer identification strategies across four biological contexts. (XLSX 176 kb)

## Data Availability

All data analyzed in this manuscript are available in the creDB R package and database (http://www.kostkalab.net/software.html).

## Abbreviations

BAR: Binding active region
BP: Biological process
CAD: Coronary artery disease
CAGE: Cap analysis of gene expression
ChIP-seq: Chromatin immunoprecipitation sequencing
creDB: Cis-REgulatory Elements DataBase
DHS: DNase I hypersensitivity site
DNase-seq: DNase I hypersensitivity site sequencing
eQTL: Expression quantitative trait loci
eRNA: Enhancer RNA
GAT: Genomic Association Tester
GO: Gene ontology
GREAT: Genomic Regions Enrichment of Annotations Tool
GRO-seq: Global run-on sequencing
GWAS: Genome-wide association study
JEME: Joint Effect of Multiple Enhancers
LD: Linkage disequilibrium
LDL: Low-density lipoprotein
MDS: Multidimensional scaling
MF: Molecular function
MI: Myocardial infarction
MPRA: Massively parallel reporter assay
TRF: Transcription-related factor
TSS: Transcription start site

## References

[1] Shlyueva Daria, Stampfel Gerald, Stark Alexander (2014). Transcriptional enhancers: from properties to genome-wide predictions. Nature Reviews Genetics.

[2] Andersson Robin, Sandelin Albin, Danko Charles G. (2015). A unified architecture of transcriptional regulatory elements. Trends in Genetics.

[3] Ong Chin-Tong, Corces Victor G. (2011). Enhancer function: new insights into the regulation of tissue-specific gene expression. Nature Reviews Genetics.

[4] Creyghton MP, Cheng AW, Welstead GG, Kooistra T, Carey BW, Steine EJ (2010). Histone H3K27ac separates active from poised enhancers and predicts developmental state. Proc Natl Acad Sci U S A.

[5] Maurano M. T., Humbert R., Rynes E., Thurman R. E., Haugen E., Wang H., Reynolds A. P., Sandstrom R., Qu H., Brody J., Shafer A., Neri F., Lee K., Kutyavin T., Stehling-Sun S., Johnson A. K., Canfield T. K., Giste E., Diegel M., Bates D., Hansen R. S., Neph S., Sabo P. J., Heimfeld S., Raubitschek A., Ziegler S., Cotsapas C., Sotoodehnia N., Glass I., Sunyaev S. R., Kaul R., Stamatoyannopoulos J. A. (2012). Systematic Localization of Common Disease-Associated Variation in Regulatory DNA. Science.

[6] Corradin O, Scacheri PC. Enhancer variants: evaluating functions in common disease. Genome Med [Internet]. 2014;6(10):85. 10.1186/s13073-014-0085-3 PMC4254432 25473424

[7] Sholtis SJ, Noonan JP. Gene regulation and the origins of human biological uniqueness. Trends Genet [Internet]. 2010;26(3):110–8. 10.1016/j.tig.2009.12.009 20106546

[8] Reilly SK, Noonan JP. Evolution of gene regulation in humans. Annu Rev Genom Hum Genet [Internet]. 2016;1–23. 10.1146/annurev-genom-090314-045935 27147089

[9] Mack Katya L., Nachman Michael W. (2017). Gene Regulation and Speciation. Trends in Genetics.

[10] Pennacchio Len A., Bickmore Wendy, Dean Ann, Nobrega Marcelo A., Bejerano Gill (2013). Enhancers: five essential questions. Nature Reviews Genetics.

[11] Kleftogiannis D, Kalnis P, Bajic VB (2016). Progress and challenges in bioinformatics approaches for enhancer identification. Brief Bioinform.

[12] Inoue F, Kircher M, Martin B, Cooper GM, Witten DM, McManus MT (2017). A systematic comparison reveals substantial differences in chromosomal versus episomal encoding of enhancer activity. Genome Res.

[13] Inoue Fumitaka, Ahituv Nadav (2015). Decoding enhancers using massively parallel reporter assays. Genomics.

[14] Heintzman Nathaniel D, Stuart Rhona K, Hon Gary, Fu Yutao, Ching Christina W, Hawkins R David, Barrera Leah O, Van Calcar Sara, Qu Chunxu, Ching Keith A, Wang Wei, Weng Zhiping, Green Roland D, Crawford Gregory E, Ren Bing (2007). Distinct and predictive chromatin signatures of transcriptional promoters and enhancers in the human genome. Nature Genetics.

[15] Hoffman MM, Ernst J, Wilder SP, Kundaje A, Harris RS, Libbrecht M (2013). Integrative annotation of chromatin elements from ENCODE data. Nucleic Acids Res.

[16] Dogan N, Wu W, Morrissey CS, Chen K-B, Stonestrom A, Long M, et al. Occupancy by key transcription factors is a more accurate predictor of enhancer activity than histone modifications or chromatin accessibility. Epigenetics Chromatin [Internet]. 2015;8(1):1–21. 10.1186/s13072-015-0009-5 PMC4432502 25984238

[17] Crawford GE, Holt IE, Whittle J, Webb BD, Tai D, Davis S (2006). Genome-wide mapping of DNase hypersensitive sites using massively parallel signature sequencing (MPSS). Genome Res.

[18] Thurman RE, Rynes E, Humbert R, Vierstra J, Maurano MT, Haugen E, et al. The accessible chromatin landscape of the human genome. Nature [Internet]. 2012;489(7414):75–82. 10.1038/nature11232 PMC3721348 22955617

[19] Heintzman Nathaniel D., Hon Gary C., Hawkins R. David, Kheradpour Pouya, Stark Alexander, Harp Lindsey F., Ye Zhen, Lee Leonard K., Stuart Rhona K., Ching Christina W., Ching Keith A., Antosiewicz-Bourget Jessica E., Liu Hui, Zhang Xinmin, Green Roland D., Lobanenkov Victor V., Stewart Ron, Thomson James A., Crawford Gregory E., Kellis Manolis, Ren Bing (2009). Histone modifications at human enhancers reflect global cell-type-specific gene expression. Nature.

[20] Woolfe Adam, Goodson Martin, Goode Debbie K, Snell Phil, McEwen Gayle K, Vavouri Tanya, Smith Sarah F, North Phil, Callaway Heather, Kelly Krys, Walter Klaudia, Abnizova Irina, Gilks Walter, Edwards Yvonne J. K, Cooke Julie E, Elgar Greg (2004). Highly Conserved Non-Coding Sequences Are Associated with Vertebrate Development. PLoS Biology.

[21] L a P, Ahituv N, Moses AM, Prabhakar S, M a N, Shoukry M (2006). In vivo enhancer analysis of human conserved non-coding sequences. Nature..

[22] Visel Axel, Prabhakar Shyam, Akiyama Jennifer A, Shoukry Malak, Lewis Keith D, Holt Amy, Plajzer-Frick Ingrid, Afzal Veena, Rubin Edward M, Pennacchio Len A (2008). Ultraconservation identifies a small subset of extremely constrained developmental enhancers. Nature Genetics.

[23] Visel Axel, Blow Matthew J., Li Zirong, Zhang Tao, Akiyama Jennifer A., Holt Amy, Plajzer-Frick Ingrid, Shoukry Malak, Wright Crystal, Chen Feng, Afzal Veena, Ren Bing, Rubin Edward M., Pennacchio Len A. (2009). ChIP-seq accurately predicts tissue-specific activity of enhancers. Nature.

[24] Blow Matthew J, McCulley David J, Li Zirong, Zhang Tao, Akiyama Jennifer A, Holt Amy, Plajzer-Frick Ingrid, Shoukry Malak, Wright Crystal, Chen Feng, Afzal Veena, Bristow James, Ren Bing, Black Brian L, Rubin Edward M, Visel Axel, Pennacchio Len A (2010). ChIP-Seq identification of weakly conserved heart enhancers. Nature Genetics.

[25] Andersson Robin, Gebhard Claudia, Miguel-Escalada Irene, Hoof Ilka, Bornholdt Jette, Boyd Mette, Chen Yun, Zhao Xiaobei, Schmidl Christian, Suzuki Takahiro, Ntini Evgenia, Arner Erik, Valen Eivind, Li Kang, Schwarzfischer Lucia, Glatz Dagmar, Raithel Johanna, Lilje Berit, Rapin Nicolas, Bagger Frederik Otzen, Jørgensen Mette, Andersen Peter Refsing, Bertin Nicolas, Rackham Owen, Burroughs A. Maxwell, Baillie J. Kenneth, Ishizu Yuri, Shimizu Yuri, Furuhata Erina, Maeda Shiori, Negishi Yutaka, Mungall Christopher J., Meehan Terrence F., Lassmann Timo, Itoh Masayoshi, Kawaji Hideya, Kondo Naoto, Kawai Jun, Lennartsson Andreas, Daub Carsten O., Heutink Peter, Hume David A., Jensen Torben Heick, Suzuki Harukazu, Hayashizaki Yoshihide, Müller Ferenc, Forrest Alistair R. R., Carninci Piero, Rehli Michael, Sandelin Albin (2014). An atlas of active enhancers across human cell types and tissues. Nature.

[26] Li W, Notani D, Rosenfeld MG (2016). Enhancers as non-coding RNA transcription units: recent insights and future perspectives. Nat Rev Genet.

[27] Young RS, Kumar Y, Bickmore WA, Taylor MS. Bidirectional transcription marks accessible chromatin and is not specific to enhancers. Genome Biol [Internet. 2017;18. 10.1186/s13059-017-1379-8 PMC5747114 29284524

[28] Arner Erik, Daub Carsten O., Vitting-Seerup Kristoffer, Andersson Robin, Lilje Berit, Drabløs Finn, Lennartsson Andreas, Rönnerblad Michelle, Hrydziuszko Olga, Vitezic Morana, Freeman Tom C., M. N. Alhendi Ahmad, Arner Peter, Axton Richard, Baillie J. Kenneth, Beckhouse Anthony, Bodega Beatrice, Briggs James, Brombacher Frank, Davis Margaret, Detmar Michael, Ehrlund Anna, Endoh Mitsuhiro, Eslami Afsaneh, Fagiolini Michela, Fairbairn Lynsey, Faulkner Geoffrey J., Ferrai Carmelo, Fisher Malcolm E., Forrester Lesley, Goldowitz Daniel, Guler Reto, Ha Thomas, Hara Mitsuko, Herlyn Meenhard, Ikawa Tomokatsu, Kai Chieko, Kawamoto Hiroshi, M. Khachigian Levon, Klinken S. Peter, Kojima Soichi, Koseki Haruhiko, Klein Sarah, Mejhert Niklas, Miyaguchi Ken, Mizuno Yosuke, Morimoto Mitsuru, Morris Kelly J., Mummery Christine, Nakachi Yutaka, Ogishima Soichi, Okada-Hatakeyama Mariko, Okazaki Yasushi, Orlando Valerio, Ovchinnikov Dmitry, Passier Robert, Patrikakis Margaret, Pombo Ana, Qin Xian-Yang, Roy Sugata, Sato Hiroki, Savvi Suzana, Saxena Alka, Schwegmann Anita, Sugiyama Daisuke, Swoboda Rolf, Tanaka Hiroshi, Tomoiu Andru, Winteringham Louise N., Wolvetang Ernst, Yanagi-Mizuochi Chiyo, Yoneda Misako, Zabierowski Susan, Zhang Peter, Abugessaisa Imad, Bertin Nicolas, Diehl Alexander D., Fukuda Shiro, Furuno Masaaki, Harshbarger Jayson, Hasegawa Akira, Hori Fumi, Ishikawa-Kato Sachi, Ishizu Yuri, Itoh Masayoshi, Kawashima Tsugumi, Kojima Miki, Kondo Naoto, Lizio Marina, Meehan Terrence F., Mungall Christopher J., Murata Mitsuyoshi, Nishiyori-Sueki Hiromi, Sahin Serkan, Nagao-Sato Sayaka, Severin Jessica, de Hoon Michiel J. L., Kawai Jun, Kasukawa Takeya, Lassmann Timo, Suzuki Harukazu, Kawaji Hideya, Summers Kim M., Wells Christine, Hume David A., Forrest Alistair R. R., Sandelin Albin, Carninci Piero, Hayashizaki Yoshihide (2015). Transcribed enhancers lead waves of coordinated transcription in transitioning mammalian cells. Science.

[29] Erwin Genevieve D., Oksenberg Nir, Truty Rebecca M., Kostka Dennis, Murphy Karl K., Ahituv Nadav, Pollard Katherine S., Capra John A. (2014). Integrating Diverse Datasets Improves Developmental Enhancer Prediction. PLoS Computational Biology.

[30] Ernst Jason, Kheradpour Pouya, Mikkelsen Tarjei S., Shoresh Noam, Ward Lucas D., Epstein Charles B., Zhang Xiaolan, Wang Li, Issner Robbyn, Coyne Michael, Ku Manching, Durham Timothy, Kellis Manolis, Bernstein Bradley E. (2011). Mapping and analysis of chromatin state dynamics in nine human cell types. Nature.

[31] Klein JC, Keith A, Agarwal V, Durham T, Shendure J (2018). Functional characterization of enhancer evolution in the primate lineage. Genome Biol.

[32] The ENCODE Project Consortium. An integrated encyclopedia of DNA elements in the human genome. Nature [Internet]. 2012;489(7414):57–74. 10.1038/nature11247 PMC3439153 22955616

[33] Harismendy Olivier, Notani Dimple, Song Xiaoyuan, Rahim Nazli G., Tanasa Bogdan, Heintzman Nathaniel, Ren Bing, Fu Xiang-Dong, Topol Eric J., Rosenfeld Michael G., Frazer Kelly A. (2011). 9p21 DNA variants associated with coronary artery disease impair interferon-γ signalling response. Nature.

[34] Rada-Iglesias Alvaro, Bajpai Ruchi, Swigut Tomek, Brugmann Samantha A., Flynn Ryan A., Wysocka Joanna (2010). A unique chromatin signature uncovers early developmental enhancers in humans. Nature.

[35] Villar Diego, Berthelot Camille, Aldridge Sarah, Rayner Tim F., Lukk Margus, Pignatelli Miguel, Park Thomas J., Deaville Robert, Erichsen Jonathan T., Jasinska Anna J., Turner James M.A., Bertelsen Mads F., Murchison Elizabeth P., Flicek Paul, Odom Duncan T. (2015). Enhancer Evolution across 20 Mammalian Species. Cell.

[36] Hay D, Hughes JR, Babbs C, Davies JOJ, Graham BJ, Hanssen LLP, et al. Genetic dissection of the α-globin super-enhancer in vivo. Nat Genet [Internet]. 2016;1–12. 10.1038/ng.3605 PMC5058437 27376235

[37] Zhernakova Daria V, Deelen Patrick, Vermaat Martijn, van Iterson Maarten, van Galen Michiel, Arindrarto Wibowo, van 't Hof Peter, Mei Hailiang, van Dijk Freerk, Westra Harm-Jan, Bonder Marc Jan, van Rooij Jeroen, Verkerk Marijn, Jhamai P Mila, Moed Matthijs, Kielbasa Szymon M, Bot Jan, Nooren Irene, Pool René, van Dongen Jenny, Hottenga Jouke J, Stehouwer Coen D A, van der Kallen Carla J H, Schalkwijk Casper G, Zhernakova Alexandra, Li Yang, Tigchelaar Ettje F, de Klein Niek, Beekman Marian, Deelen Joris, van Heemst Diana, van den Berg Leonard H, Hofman Albert, Uitterlinden André G, van Greevenbroek Marleen M J, Veldink Jan H, Boomsma Dorret I, van Duijn Cornelia M, Wijmenga Cisca, Slagboom P Eline, Swertz Morris A, Isaacs Aaron, van Meurs Joyce B J, Jansen Rick, Heijmans Bastiaan T, 't Hoen Peter A C, Franke Lude (2016). Identification of context-dependent expression quantitative trait loci in whole blood. Nature Genetics.

[38] Weinhold Nils, Jacobsen Anders, Schultz Nikolaus, Sander Chris, Lee William (2014). Genome-wide analysis of noncoding regulatory mutations in cancer. Nature Genetics.

[39] Core LJ, Martins AL, Danko CG, Waters CT, Siepel A, Lis JT. Analysis of nascent RNA identifies a unified architecture of initiation regions at mammalian promoters and enhancers. Nat Genet. 2014. 10.1038/ng.3142 PMC4254663 25383968

[40] Parker SCJ, Stitzel ML, Taylor DL, Orozco JM, Erdos MR, Akiyama JA, et al. Chromatin stretch enhancer states drive cell-specific gene regulation and harbor human disease risk variants. Proc Natl Acad Sci U S A [Internet]. 2013;110(44):17921–926. 10.1073/pnas.1317023110 PMC3816444 24127591

[41] Gusev A, Lee SH, Trynka G, Finucane H, Vilhjálmsson BJ, Xu H (2014). Partitioning heritability of regulatory and cell-type-specific variants across 11 common diseases. Am J Hum Genet.

[42] Onengut-Gumuscu Suna, Chen Wei-Min, Burren Oliver, Cooper Nick J, Quinlan Aaron R, Mychaleckyj Josyf C, Farber Emily, Bonnie Jessica K, Szpak Michal, Schofield Ellen, Achuthan Premanand, Guo Hui, Fortune Mary D, Stevens Helen, Walker Neil M, Ward Lucas D, Kundaje Anshul, Kellis Manolis, Daly Mark J, Barrett Jeffrey C, Cooper Jason D, Deloukas Panos, Todd John A, Wallace Chris, Concannon Patrick, Rich Stephen S (2015). Fine mapping of type 1 diabetes susceptibility loci and evidence for colocalization of causal variants with lymphoid gene enhancers. Nature Genetics.

[43] Yip Kevin Y, Cheng Chao, Bhardwaj Nitin, Brown James B, Leng Jing, Kundaje Anshul, Rozowsky Joel, Birney Ewan, Bickel Peter, Snyder Michael, Gerstein Mark (2012). Classification of human genomic regions based on experimentally determined binding sites of more than 100 transcription-related factors. Genome Biology.

[44] Ho JWK, Jung YL, Liu T, Alver BH, Lee S, Ikegami K, et al. Comparative analysis of metazoan chromatin organization. Nature [Internet]. 2014;512(7515):449–52. 10.1038/nature13415 PMC4227084 25164756

[45] Siepel A, Bejerano G, Pedersen JS, Hinrichs AS, Hou M, Rosenbloom K (2005). Evolutionarily conserved elements in vertebrate, insect, worm, and yeast genomes. Genome Res.

[46] Visel A, Minovitsky S, Dubchak I, Pennacchio LAVISTA (2007). Enhancer browser - a database of tissue-specific human enhancers. Nucleic Acids Res.

[47] Ernst J, Melnikov A, Zhang X, Wang L, Rogov P, Mikkelsen TS (2016). Genome-scale high-resolution mapping of activating and repressive nucleotides in regulatory regions. Nat Biotechnol.

[48] Ward Lucas D, Kellis Manolis (2012). Interpreting noncoding genetic variation in complex traits and human disease. Nature Biotechnology.

[49] Edwards Stacey L., Beesley Jonathan, French Juliet D., Dunning Alison M. (2013). Beyond GWASs: Illuminating the Dark Road from Association to Function. The American Journal of Human Genetics.

[50] Ashoor H, Kleftogiannis D, Radovanovic A, Bajic VB. DENdb: database of integrated human enhancers. Database. 2015;2015. 10.1093/database/bav085 PMC4560934 26342387

[51] Gao T, He B, Liu S, Zhu H, Tan K, Qian J. EnhancerAtlas: a resource for enhancer annotation and analysis in 105 human cell/tissue types. Bioinformatics [Internet]. 2016;(August):btw495. 10.1093/bioinformatics/btw495 PMC5181530 27515742

[52] Pasquali L, Gaulton KJ, Rodriguez-Segui SA, Mularoni L, Miguel-Escalada I, Akerman I (2014). Pancreatic islet enhancer clusters enriched in type 2 diabetes risk-associated variants. Nat Genet.

[53] Hazelett DJ, Rhie SK, Gaddis M, Yan C, Lakeland DL, Coetzee SG (2014). Comprehensive functional annotation of 77 prostate Cancer risk loci. PLoS Genet.

[54] Farh Kyle Kai-How, Marson Alexander, Zhu Jiang, Kleinewietfeld Markus, Housley William J., Beik Samantha, Shoresh Noam, Whitton Holly, Ryan Russell J. H., Shishkin Alexander A., Hatan Meital, Carrasco-Alfonso Marlene J., Mayer Dita, Luckey C. John, Patsopoulos Nikolaos A., De Jager Philip L., Kuchroo Vijay K., Epstein Charles B., Daly Mark J., Hafler David A., Bernstein Bradley E. (2014). Genetic and epigenetic fine mapping of causal autoimmune disease variants. Nature.

[55] Musunuru K, Strong A, Frank-Kamenetsky M, Lee NE, Ahfeldt T, Sachs KV, et al. From noncoding variant to phenotype via SORT1 at the 1p13 cholesterol locus. Nature. 2010. 10.1038/nature09266 PMC3062476 20686566

[56] Almontashiri NAM, Antoine D, Zhou X, Vilmundarson RO, Zhang SX, Hao KN, et al. 9p21.3 coronary artery disease risk variants disrupt TEAD transcription factor-dependent transforming growth factor β regulation of p16 expression in human aortic smooth muscle cells. Circulation. 2015. 10.1161/CIRCULATIONAHA.114.015023 26487755

[57] McLean Cory Y, Bristor Dave, Hiller Michael, Clarke Shoa L, Schaar Bruce T, Lowe Craig B, Wenger Aaron M, Bejerano Gill (2010). GREAT improves functional interpretation of cis-regulatory regions. Nature Biotechnology.

[58] Cao Q, Anyansi C, Hu X, Xu L, Xiong L, Tang W (2017). Reconstruction of enhancer-target networks in 935 samples of human primary cells, tissues and cell lines. Nat Genet.

[59] Wang J. Z., Du Z., Payattakool R., Yu P. S., Chen C.-F. (2007). A new method to measure the semantic similarity of GO terms. Bioinformatics.

[60] Yu G, Li F, Qin Y, Bo X, Wu Y, Wang S (2010). GOSemSim: an R package for measuring semantic similarity among GO terms and gene products. Bioinformatics..

[61] Weedon MN, Cebola I, Patch A-M, Flanagan SE, De Franco E, Caswell R (2014). Recessive mutations in a distal PTF1A enhancer cause isolated pancreatic agenesis. Nat Genet.

[62] Yao L, Tak YG, Berman BP, Farnham PJ. Functional annotation of colon cancer risk SNPs. Nat Commun [Internet]. 2014;5:5114. 10.1038/ncomms6114 PMC4200523 25268989

[63] Tak YG, Farnham PJ (2015). Making sense of GWAS: using epigenomics and genome engineering to understand the functional relevance of SNPs in non-coding regions of the human genome. Epigenetics Chromatin.

[64] Chatterjee S, Ahituv N. Gene regulatory elements , major drivers of human disease. Annu Rev Genom Hum Genet. 2017:1–19. 10.1146/annurev-genom-091416-035537 28399667

[65] McVicker G, Van De Geijn B, Degner JF, Cain CE, Banovich NE, Raj A (2013). Identification of genetic variants that affect histone modifications in human cells. Science (80- ).

[66] Kasowski M., Kyriazopoulou-Panagiotopoulou S., Grubert F., Zaugg J. B., Kundaje A., Liu Y., Boyle A. P., Zhang Q. C., Zakharia F., Spacek D. V., Li J., Xie D., Olarerin-George A., Steinmetz L. M., Hogenesch J. B., Kellis M., Batzoglou S., Snyder M. (2013). Extensive Variation in Chromatin States Across Humans. Science.

[67] Taudt Aaron, Colomé-Tatché Maria, Johannes Frank (2016). Genetic sources of population epigenomic variation. Nature Reviews Genetics.

[68] Catarino RR, Stark A (2018). Assessing sufficiency and necessity of enhancer activities for gene expression and the mechanisms of transcription activation. Genes Dev.

[69] Dao Lan T M, Galindo-Albarrán Ariel O, Castro-Mondragon Jaime A, Andrieu-Soler Charlotte, Medina-Rivera Alejandra, Souaid Charbel, Charbonnier Guillaume, Griffon Aurélien, Vanhille Laurent, Stephen Tharshana, Alomairi Jaafar, Martin David, Torres Magali, Fernandez Nicolas, Soler Eric, van Helden Jacques, Puthier Denis, Spicuglia Salvatore (2017). Genome-wide characterization of mammalian promoters with distal enhancer functions. Nature Genetics.

[70] Pradeepa Madapura M, Grimes Graeme R, Kumar Yatendra, Olley Gabrielle, Taylor Gillian C A, Schneider Robert, Bickmore Wendy A (2016). Histone H3 globular domain acetylation identifies a new class of enhancers. Nature Genetics.

[71] Zhou J, Troyanskaya OG. Probabilistic modelling of chromatin code landscape reveals functional diversity of enhancer-like chromatin states. Nat Commun [Internet]. 2016;7:1–9. 10.1038/ncomms10528 PMC4742914 26841971

[72] Kim TK, Shiekhattar R (2015). Architectural and functional commonalities between enhancers and promoters. Cell..

[73] Andersson R (2015). Promoter or enhancer, what’s the difference? Deconstruction of established distinctions and presentation of a unifying model. BioEssays..

[74] Roadmap Epigenomics Consortium, Kundaje A, Meuleman W, Ernst J, Bilenky M, Yen A, et al. Integrative analysis of 111 reference human epigenomes. Nature [Internet]. 2015;518(7539):317–30. 10.1038/nature14248 PMC4530010 25693563

[75] Ernst Jason, Kellis Manolis (2012). ChromHMM: automating chromatin-state discovery and characterization. Nature Methods.

[76] Kundaje A. A comprehensive collection of signal artifact blacklist regions in the human genome. Site/Anshulkundaje/Projects/Blacklists (Last Accessed 30 [Internet]. 2013; Available from: https://sites.google.com/site/anshulkundaje/projects/blacklists.

[77] Li H, Durbin R (2009). Fast and accurate short read alignment with burrows-wheeler transform. Bioinformatics..

[78] Zhang Yong, Liu Tao, Meyer Clifford A, Eeckhoute Jérôme, Johnson David S, Bernstein Bradley E, Nussbaum Chad, Myers Richard M, Brown Myles, Li Wei, Liu X Shirley (2008). Model-based Analysis of ChIP-Seq (MACS). Genome Biology.

[79] Welter Danielle, MacArthur Jacqueline, Morales Joannella, Burdett Tony, Hall Peggy, Junkins Heather, Klemm Alan, Flicek Paul, Manolio Teri, Hindorff Lucia, Parkinson Helen (2013). The NHGRI GWAS Catalog, a curated resource of SNP-trait associations. Nucleic Acids Research.

[80] The GTEx Consortium. The genotype-tissue expression (GTEx) project. Nat Genet [Internet]. 2013;45(6):580–85. 10.1038/ng.2653 PMC4010069 23715323

[81] 1000 Genomes Project Consortium, Auton A, Brooks LD, Durbin RM, Garrison EP, Kang HM, et al. A global reference for human genetic variation. Nature [Internet]. 2015;526(7571):68–74. 10.1038/nature15393 PMC4750478 26432245

[82] Quinlan AR, Hall IM (2010). BEDTools: a flexible suite of utilities for comparing genomic features. Bioinformatics..

[83] Wickham H. ggplot2 [Internet]. Elegant Graphics for Data Analysis. 2009. p. 221

[84] Heger A, Webber C, Goodson M, Ponting CP, Lunter G (2013). GAT: a simulation framework for testing the association of genomic intervals. Bioinformatics..

[85] Wang J, Vasaikar S, Shi Z, Greer M, Zhang B (2017). WebGestalt 2017: a more comprehensive, powerful, flexible and interactive gene set enrichment analysis toolkit. Nucleic Acids Res.

[86] McLeay RC, Bailey TL. Motif enrichment analysis: a unified framework and an evaluation on ChIP data. BMC Bioinformatics. 2010. 10.1186/1471-2105-11-165 PMC2868005 20356413

[87] Kulakovskiy IV, Vorontsov IE, Yevshin IS, Sharipov RN, Fedorova AD, Rumynskiy EI, et al. HOCOMOCO: towards a complete collection of transcription factor binding models for human and mouse via large-scale ChIP-Seq analysis. Nucleic Acids Res. 2018. 10.1093/nar/gkx1106 PMC5753240 29140464

[88] R Core Team. R Core Team (2015). R: a language and environment for statistical computing. R found stat Comput Vienna, Austria. R Foundation for Statistical Computing; 2015. http://www.Rproject.org/

[89] Galili T (2015). Dendextend: an R package for visualizing, adjusting and comparing trees of hierarchical clustering. Bioinformatics..

