# Supplementary material for: Genome-wide enhancer annotations differ significantly in genomic distribution, evolution, and function
Source: BMC Genomics. 2019 Jun 20;20:511. doi: 10.1186/s12864-019-5779-x (PMC6585034; doi:10.1186/s12864-019-5779-x)
Supplement: Supplementary file 1 — Figure S1. Enhancer sets across all contexts considered differ in both number (A) and length (B): Gm12878; Heart. Figure S2. Enhancer sets have low amounts of overlap with each other in bp-wise comparisons. Figure S3. Enhancer sets overlap more than expected by chance in element-wise comparisons. Figure S4. Enhancer sets have low amounts of overlap with each other in element-wise comparisons. Figure S5. Enhancers identified by different methods differ in enrichment for base pair overlap with functional attributes. Figure S6. Enhancer identification strategies recognize different subsets of validated enhancers. Figure S7. K562 enhancer sets have similar low levels of enrichment for activating regions validated by Sharpr-MPRA. Figure S8. Even among the variants in each functional LD block (r2 > 0.9) with the most enhancer set overlap, there is substantial disagreement between enhancer identification methods. Figure S9. Pairwise similarity for GO Molecular Function (MF) enrichments for enhancer sets based on JEME’s putative mappings to target genes in K562 (A), Gm12878 (B), liver (C), and heart (D). Figure S10. Pairwise similarity for GO Biological Process (BP) for enhancer sets based on JEME’s putative mappings to target genes in K562 (A), Gm12878 (B), liver (C), and heart (D). Figure S11. Pairwise similarity for GO Molecular Function (MF) enrichments from GREAT for liver enhancer sets. Figure S12. There is low pairwise similarity between GO Molecular Function (MF) enrichments calculated with GREAT for enhancer sets in the same context. Figure S13. There is low pairwise similarity between GO Biological Process (BP) enrichments calculated with GREAT for enhancer sets in the same context. Figure S14. Clustering enhancer sets on similarity of enriched transcription factor binding motifs illustrates different clustering of methods. Figure S15. Regions identified as enhancers by multiple methods do not have higher confidence scores than regions identified by a single metho [file 12864_2019_5779_MOESM1_ESM.docx]

**Supplemental Information**

**Figure S1.** Enhancer sets across all contexts considered differ in both number (A) and length (B): Gm12878; Heart.

**Figure S2.** Enhancer sets have low amounts of overlap with each other in bp-wise comparisons. The heatmaps show the percentage of element overlaps between pairs of enhancer sets in (A) Gm12878, and (B) heart. The lower heatmaps show the Jaccard similarity and the relative Jaccard similarity for (C) Gm12878 and (D) heart.

**Figure S3.** Enhancer sets overlap more than expected by chance in element-wise comparisons. Boxplots show the enrichment of element-wise overlap relative to randomly distributed regions for each pair of enhancer sets stratified by context. Only contexts with annotations across all biological contexts are included. As in the bp-wise comparisons, liver and heart enhancer sets had lower enrichments.

**Figure S4.** Enhancer sets have low amounts of overlap with each other in element-wise comparisons. The heatmap shows the percentage of element overlaps between pairs of enhancer sets in (A) K562, (B) Gm12878, (C) liver, and (D) heart. Since there are far fewer elements in a given enhancer set than there are base pairs and we did not restrict the required amount of overlap, these percentages are higher than their base-pair-wise counterparts. Nonetheless, even with more lenient element overlap criterion, we still see low percentages overall (mean 25-34% element-wise overlap; Table S2).

**Figure S5.** Enhancers identified by different methods differ in enrichment for base pair overlap with functional attributes.

(A) Enhancer sets vary in their degree of evolutionary conservation. Each point represents the enrichment for base pair (bp) overlap between each enhancer set and a primate or vertebrate PhastCons conserved element compared to randomly shuffled genomic regions. GRO-cap, FANTOM, and Yip12 are the most conserved (~3-4-fold enrichment), while sets based primarily on histone modification data are among the least conserved (~1.4–1.8 fold enrichment). (B) GWAS SNP enrichment among all enhancer sets for each biological context. All sets except FANTOM in K562 (FC = 0.8; not shown) and FANTOM in liver are significantly enriched. (C) There is some GTEx eQTL enrichment among all enhancer sets for each biological context. Enrichment for overlap with eQTL is similar to that of GWAS SNPs, except in FANTOM. DNasePlusHistone enrichment in heart is modest and not statistically significant (FC = 1.03).

**
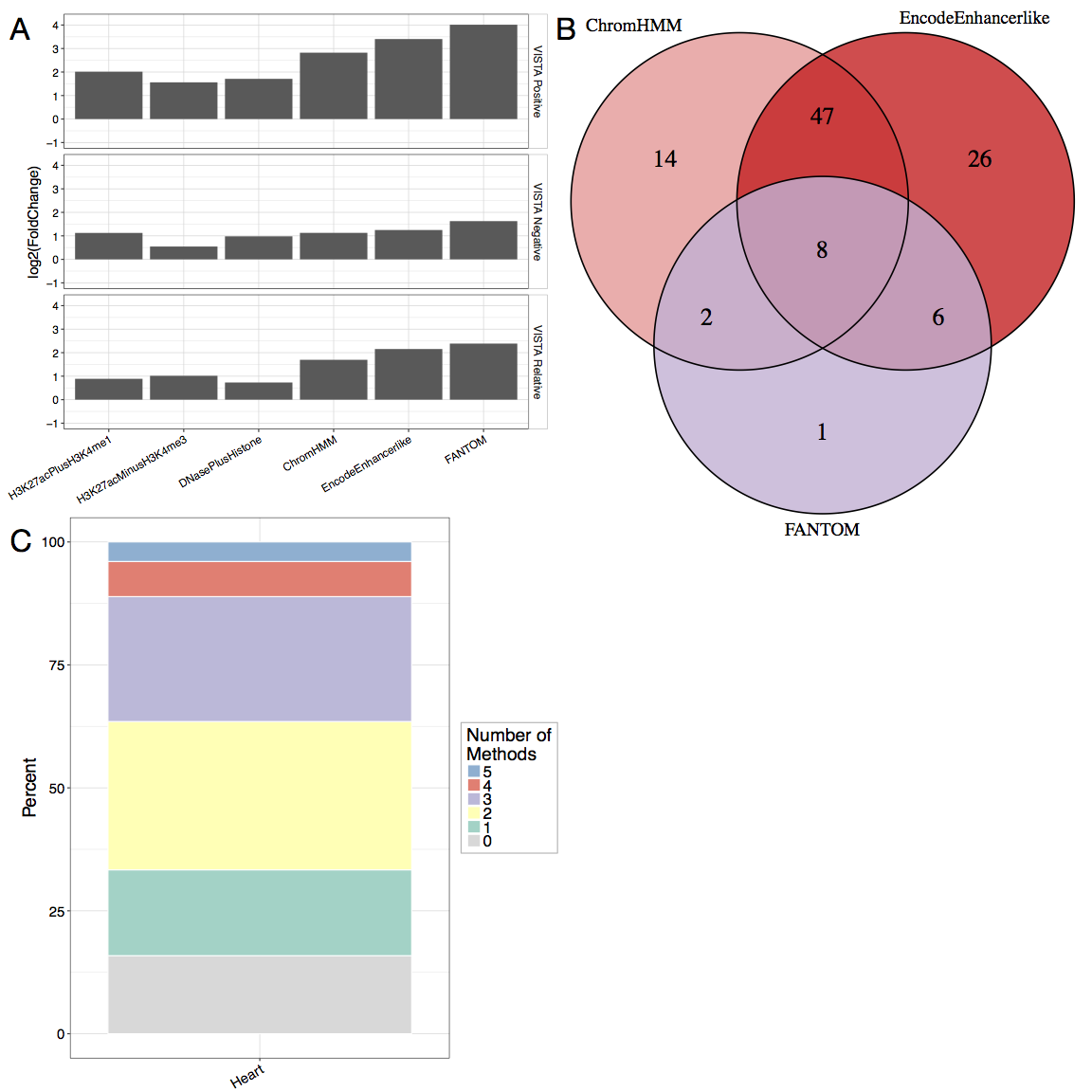
**

**Figure S6.** Enhancer identification strategies recognize different subsets of validated enhancers. (A) Plot of the element-wise enrichment for 126 positive VISTA heart enhancers (upper panel) and 882 negative VISTA regions (middle panel). All heart enhancer sets are significantly enriched for overlap with the VISTA positives (p < 0.001 for all), and each set is at least ~3x more likely to overlap validated enhancers than expected if it was randomly distributed across the genome. The FANTOM set is 16x enriched; however, given its smaller size, this was based on 17 overlaps compared to an expected ~0. All heart sets are also significantly enriched for overlap with VISTA negatives (p <= 0.004). This is not surprising as the regions tested by VISTA were largely selected based on some evidence of enhancer activity, and they may have enhancer activity in other contexts not tested by VISTA. To evaluate the ability of different methods to distinguish VISTA positives from negatives, we computed the relative enrichment for VISTA positives vs. negatives. The bottom panel is the log_2_ of the relative enrichment ratio for heart enhancer sets with VISTA heart positives compared to VISTA negatives. A positive value indicates higher enrichment for VISTA enhancers with activity in the heart, and a negative value indicates more enrichment for non-enhancers tested by VISTA. Equal enrichment in both sets yields a score of 0. The methods in heart demonstrate some ability to distinguish between the positives and negatives; however, there are only small differences in relative enrichment between the histone derived enhancer sets. Overall, FANTOM heart enhancers have the highest enrichment for experimentally validated enhancers relative to the negative set, but again we note that the FANTOM results are based on relatively small numbers of enhancers (Table S3). (B) Nearly 40% (41/104) of the VISTA heart positives identified by the top three enriched methods are unique to one method. (C) Out of the validated VISTA heart enhancers, and 17% are identified by a single method. Less than 5% of the positives are identified by all methods. This suggests that different methods identify different subsets of validated enhancers.

**Figure S7.** K562 enhancer sets have similar low levels of enrichment for activating regions validated by Sharpr-MPRA. (A) Enrichment (log_2_ fold change) for activating (top) and repressive (middle) regions as defined by the Sharpr-MPRA assay in K562. All of the K562 enhancer sets are significantly enriched for overlap with both activating and repressive regions (p < 0.001 for all). There is little variation between the methods, with most being ~10x enriched; GRO-cap has the highest enrichment (26x; p < 0.001) followed by FANTOM (17x; p < 0.001). The bottom panel shows the relative enrichment for activating regions of each enhancer set. The relative enrichment is low and generally consistent across methods. (B) Many of the MPRA validated regions are not shared between the top three enriched sets (FANTOM, p300, GRO-cap). Among the activating regions identified by the top three methods, 69% are unique to a single set and only 25 are shared by all three. (C) In general, nearly half of the MPRA activating regions were not identified by any of the enhancer sets (47%, 2,508 / 5,373), and 30% of activating elements overlapped by at least one enhancer set are unique to a single set (819 / 2,747). This suggests that, like VISTA, different strategies may identify different subsets of regulatory regions identified as active in the same context by the MPRA. We note that ChromHMM enhancer states and DNase I hypersensitivity data were used to generate the catalog of regions tested in the MPRA. This biases these results, and thus, we caution against directly comparing the enrichment of different enhancer sets. Additionally, like lower-throughput reporter assays, MPRA approaches also suffer from inaccuracies induced by experimental variation, length restrictions on the tested sequences, and removal of the tested element from its endogenous context (Inoue and Ahituv 2015).

**Figure S8.** Even among the variants in each functional LD block (r^2^ > 0.9) with the most enhancer set overlap, there is substantial disagreement between enhancer identification methods. (A) Liver enhancer sets are less enriched for variants in LD with GWAS tag SNPs than with the tag SNPs, but more importantly, a similar magnitude of enrichment is observed across enhancer identification methods. Transparent points indicate non-significant enrichment values. (B) For all GWAS SNPs, the colored bars represent the number of methods that identified the region as an enhancer in K562 or Gm12878. Few GWAS variants are shared among all methods. (C) As in the GWAS SNP analysis, we calculated the number of GTEx eQTL that overlap putative enhancers in K562 and Gm12878; the majority is supported by zero to one method.


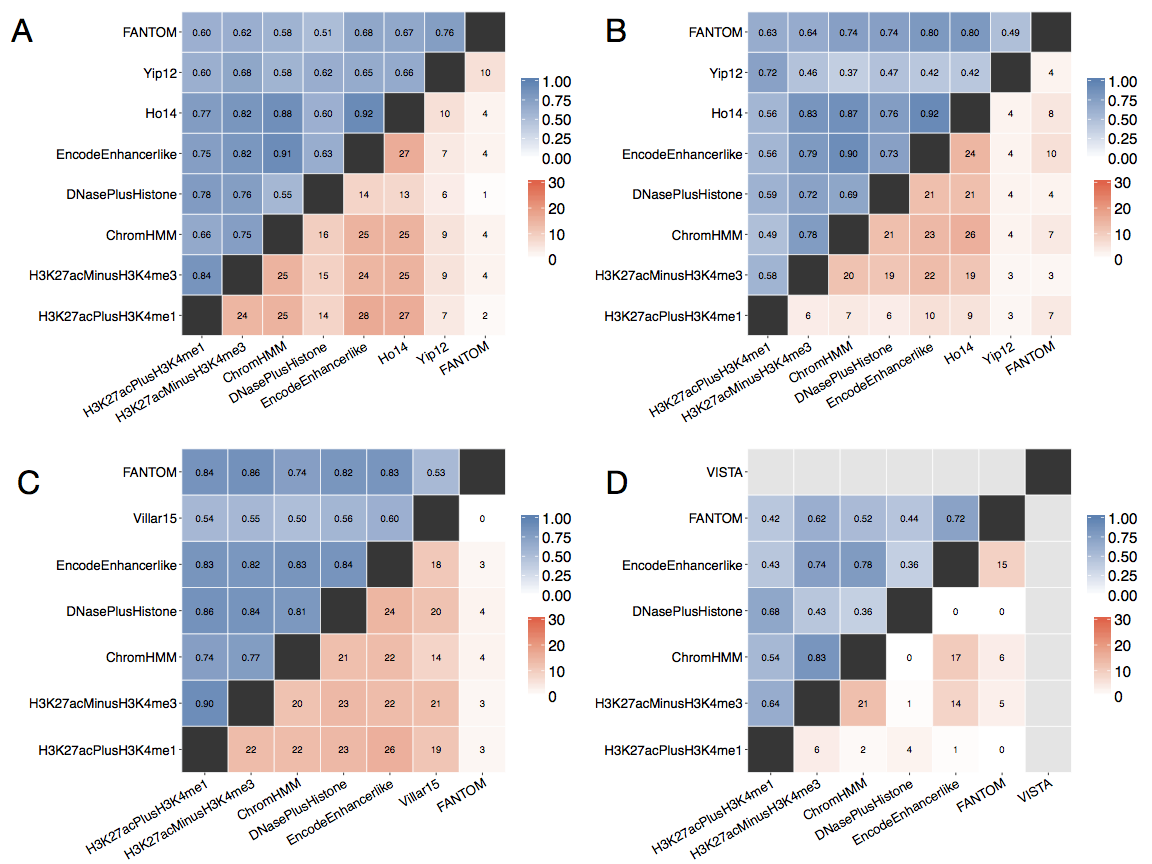


**Figure S9.** Pairwise similarity for GO Molecular Function (MF) enrichments for enhancer sets based on JEME’s putative mappings to target genes in K562 (A), Gm12878 (B), liver (C), and heart (D). The upper triangle shows the semantic similarity calculated using GoSemSim, and the lower triangle shows the number of shared terms of the top 30 most significantly enriched. Gray squares indicate that the analysis found no significantly enriched terms. This analysis does not include p300 or GRO-cap data for K562 or Gm12878. There is greater similarity between these associations compared to GREAT (Figure 5E; Figure S12), although the similarity remains relatively low and many of the matched terms are high in the hierarchy.

**
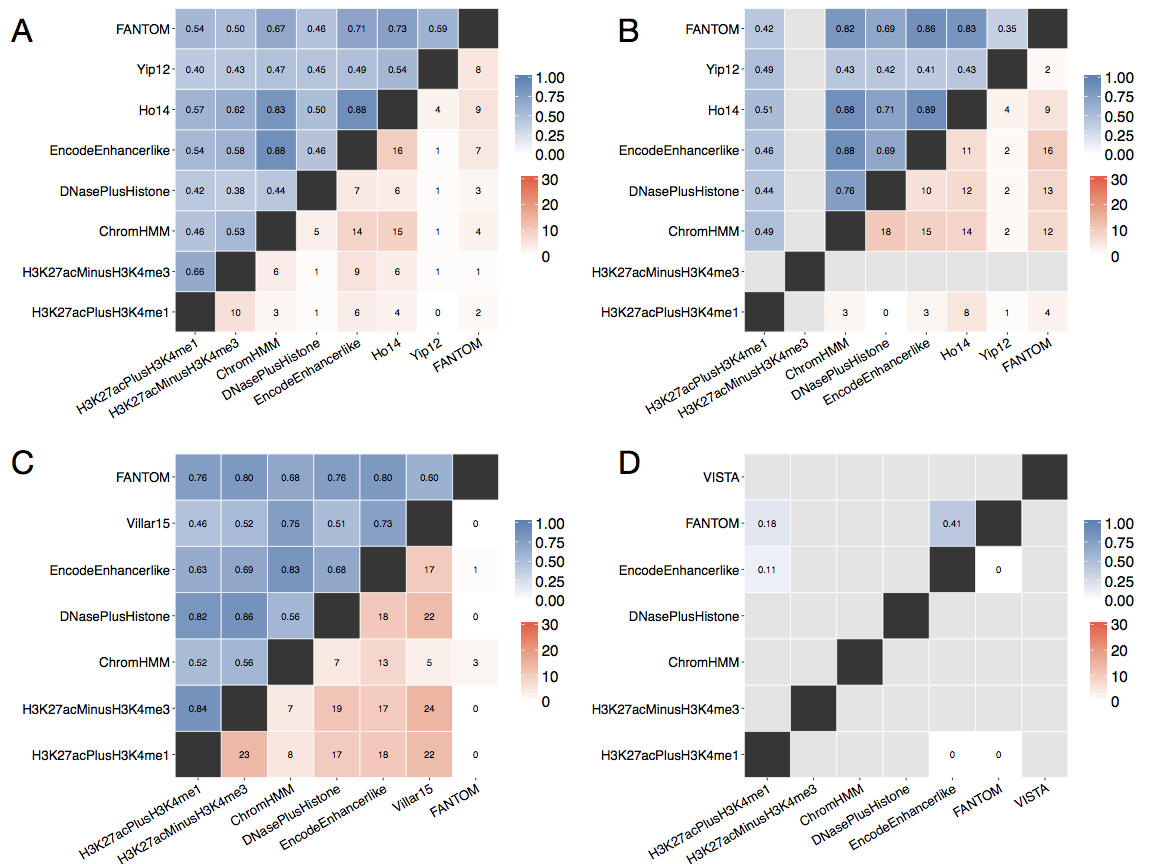
 Figure S10.** Pairwise similarity for GO Biological Process (BP) for enhancer sets based on JEME’s putative mappings to target genes in K562 (A), Gm12878 (B), liver (C), and heart (D). The upper triangle shows the semantic similarity calculated using GoSemSim, and the lower triangle shows the number of shared terms of the top 30 most significantly enriched. Gray squares indicate that the analysis found no significantly enriched terms. This analysis does not include p300 or GRO-cap data for K562 or Gm12878. Scores for the BP ontology are noticeably lower than those for the MF ontology (Figure S13). There are few significantly enriched terms for genes mapped from heart enhancers.

**
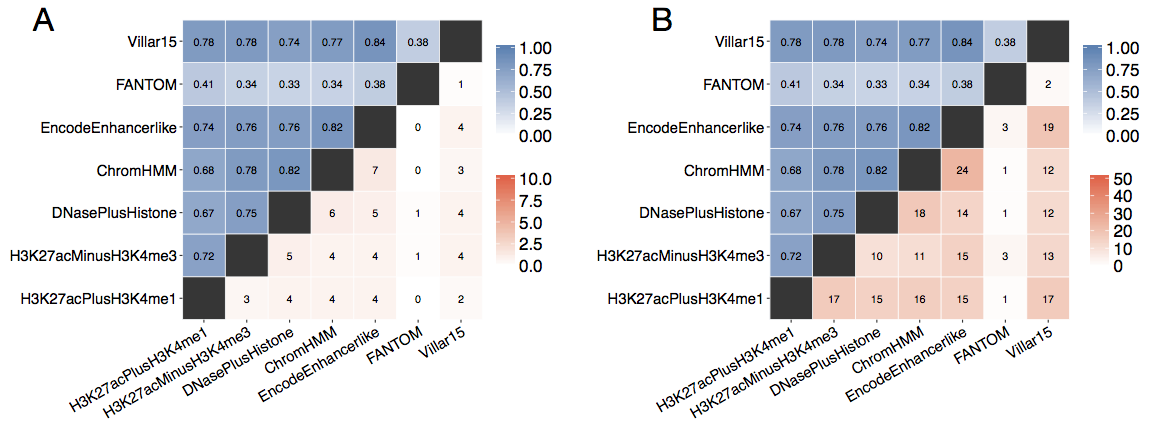
**

**Figure S11.** Pairwise similarity for GO Molecular Function (MF) enrichments from GREAT for liver enhancer sets. The upper triangle shows the semantic similarity from GoSemSim; the lower triangle shows the number of top 10 (A) and top 50 (B) most significant GO MF terms shared by each pair of enhancer sets. The majority of pairwise comparisons share far fewer than half of the significant terms.

**Figure S12.** There is low pairwise similarity between GO Molecular Function (MF) enrichments calculated with GREAT for enhancer sets in the same context. The upper triangle shows the semantic similarity from GoSemSim; the lower triangle shows the number of top 30 most significant GO MF terms shared by each pair of enhancer sets in K562 (A), Gm12878 (B), and heart (C).

**
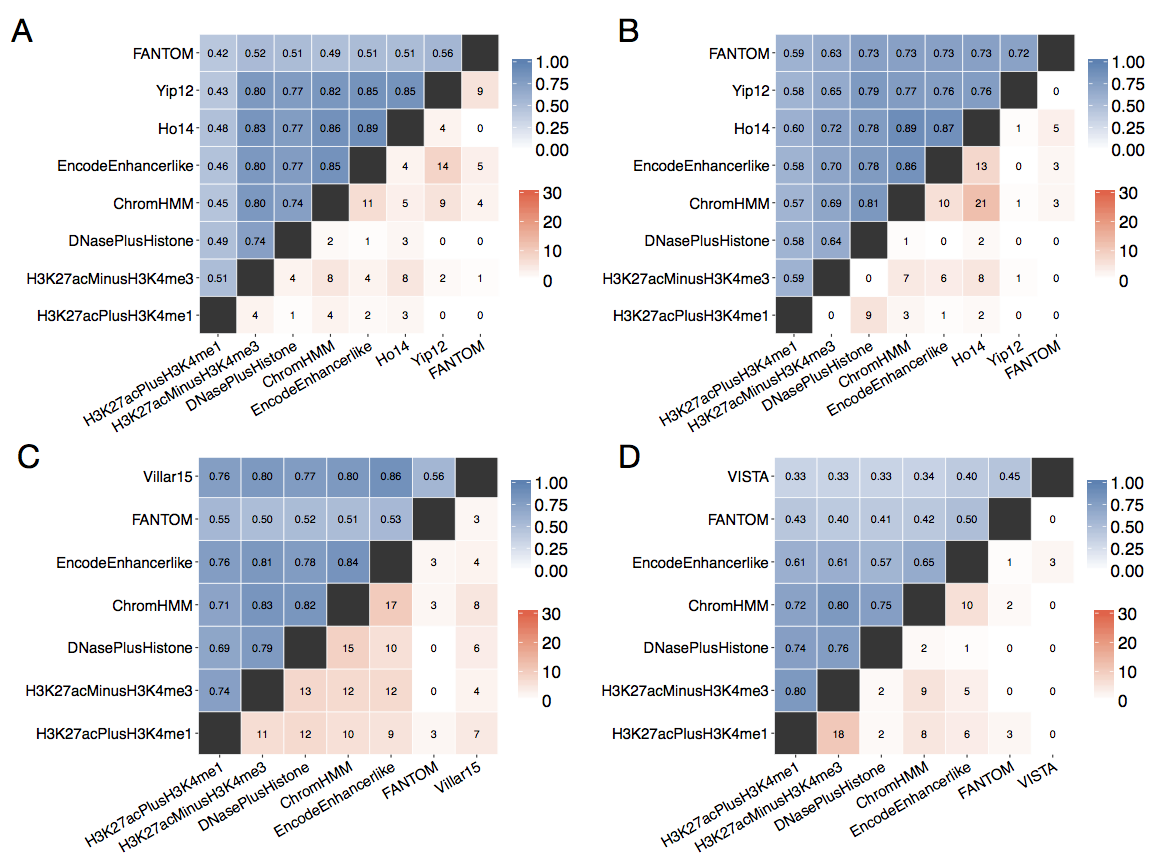
**

**Figure S13.** There is low pairwise similarity between GO Biological Process (BP) enrichments calculated with GREAT for enhancer sets in the same context. Pairwise similarity for GO MF terms for enhancer sets in K562 (A), Gm12878 (B), liver (C), and heart (D). The upper triangle shows the semantic similarity calculated using GoSemSim, and the lower triangle shows the number of shared terms of the top 30 most significantly enriched. This analysis does not include p300 or GRO-cap data for K562 or Gm12878. Scores for the BP ontology are noticeably lower than those for the MF ontology. There are few significantly enriched terms for genes mapped from heart enhancers.

**Figure S14.** Clustering enhancer sets on similarity of enriched transcription factor binding motifs illustrates different clustering of methods. We computed the enrichment of 402 HOCOMOCO (v11) core TF binding site motifs in different enhancer sets with the Analysis of Motif Enrichment (AME) tool from the MEME suite (62,63). We then clustered the TF motif enrichment profiles for each enhancer set based on Jaccard similarities using multidimensional scaling (MDS) (Methods). FANTOM and GRO-cap are consistent outliers with the largest differences in predicted TF binding site enrichment, as in the genomic and GO analyses reported in Figure 6A-B; however, the clustering of other methods varies. Most enhancer sets are enriched for more than half of the motifs in the database (~300) compared to dinucleotide frequency matched random sequences, and thus most pairs of sets have Jaccard similarity >0.8. Random motif sets of a matched size produce average Jaccard similarities between 0.6–0.69. However, we note that due to the context-dependent nature of transcription factor binding, the presence or enrichment of a motif does not guarantee function(25,64). Alternately, a lack of a significant enrichment for a binding site does not necessarily indicate a lack of activity. When combined with the observed dissimilarity of GO terms, these results suggest that similarities in sequence-level characteristics may not translate into similar regulatory targets. (A) K562, (B) Gm12878, (C) liver, and (D) heart.


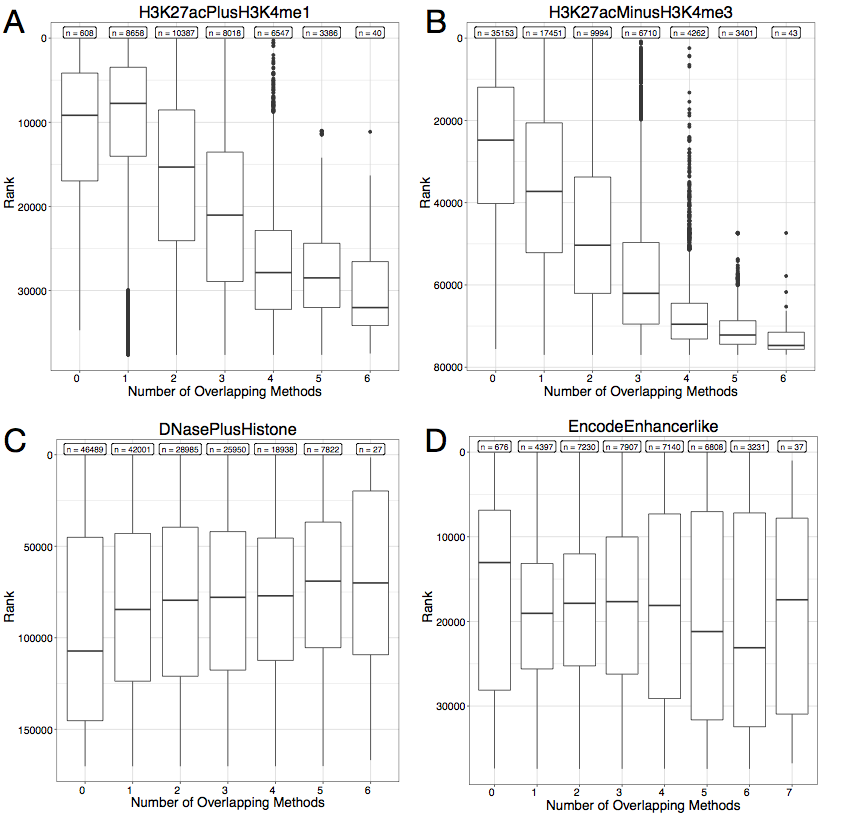


**Figure S15.** Regions identified as enhancers by multiple methods do not have higher confidence scores than regions identified by a single method. The confidence distributions for regions shared between multiple enhancer sets are similar to the confidence distributions of regions unique to a single set: (A) H3K27acPlusH3K4me1, (B) H3K27acMinusH3K4me3, (C) DNasePlusHistone, and (D) EncodeEnhancerlike. In some cases (A-B) the median score decreases as the regions are shared by more methods. While possibly a sign of poor specificity in shared enhancer regions, this trend may also be explained by transcription factor binding within or near histone acetylation sites. Transcription factor binding has been previously associated with the local minima of acetylation ChIP-seq binding profiles, so the decrease in peak signals may indicate that some of these shared regions are correlated with TF binding activity (83).


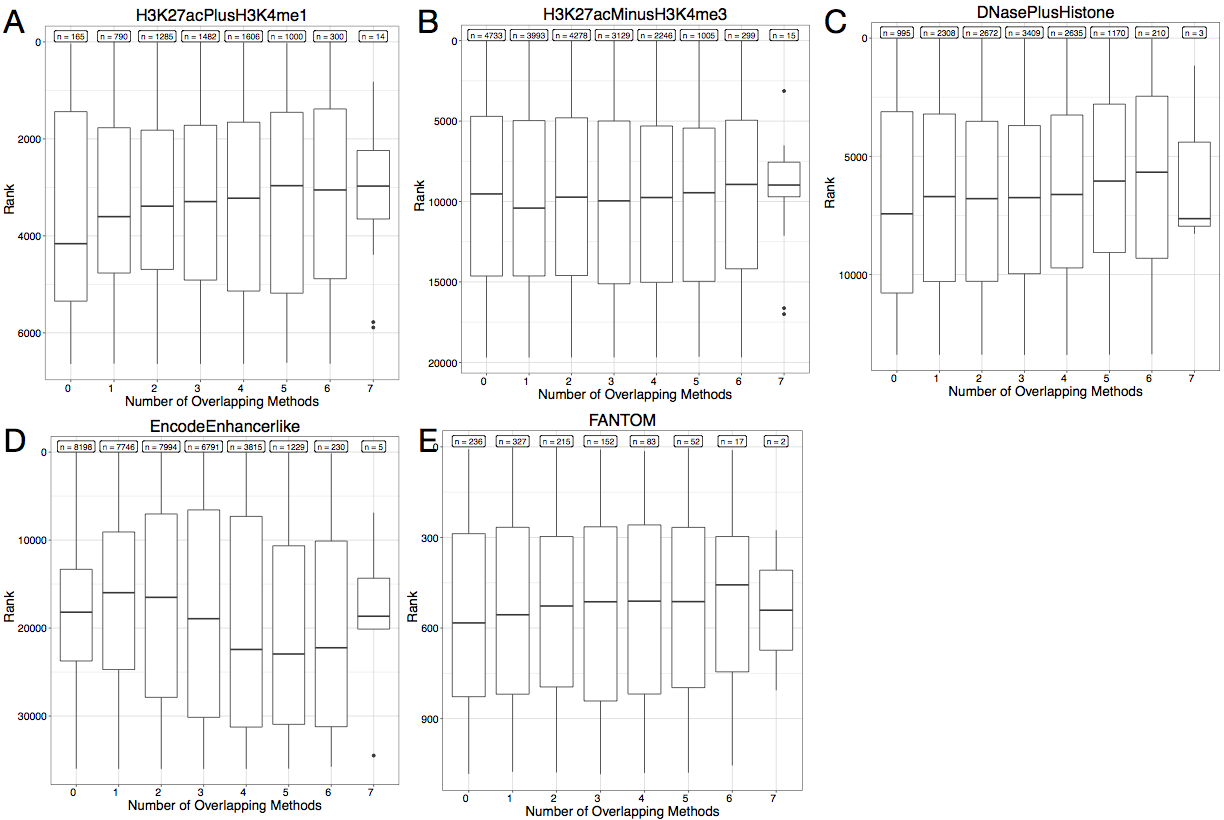


**Figure S16.** Score distributions for K562 enhancer sets are similar between regions identified as enhancers by a single method and those identified by multiple methods: (A) H3K27acPlusH3K4me1, (B) H3K27acMinusH3K4me3, (C) DNasePlusHistone, (D) EncodeEnhancerlike, and (E) FANTOM.


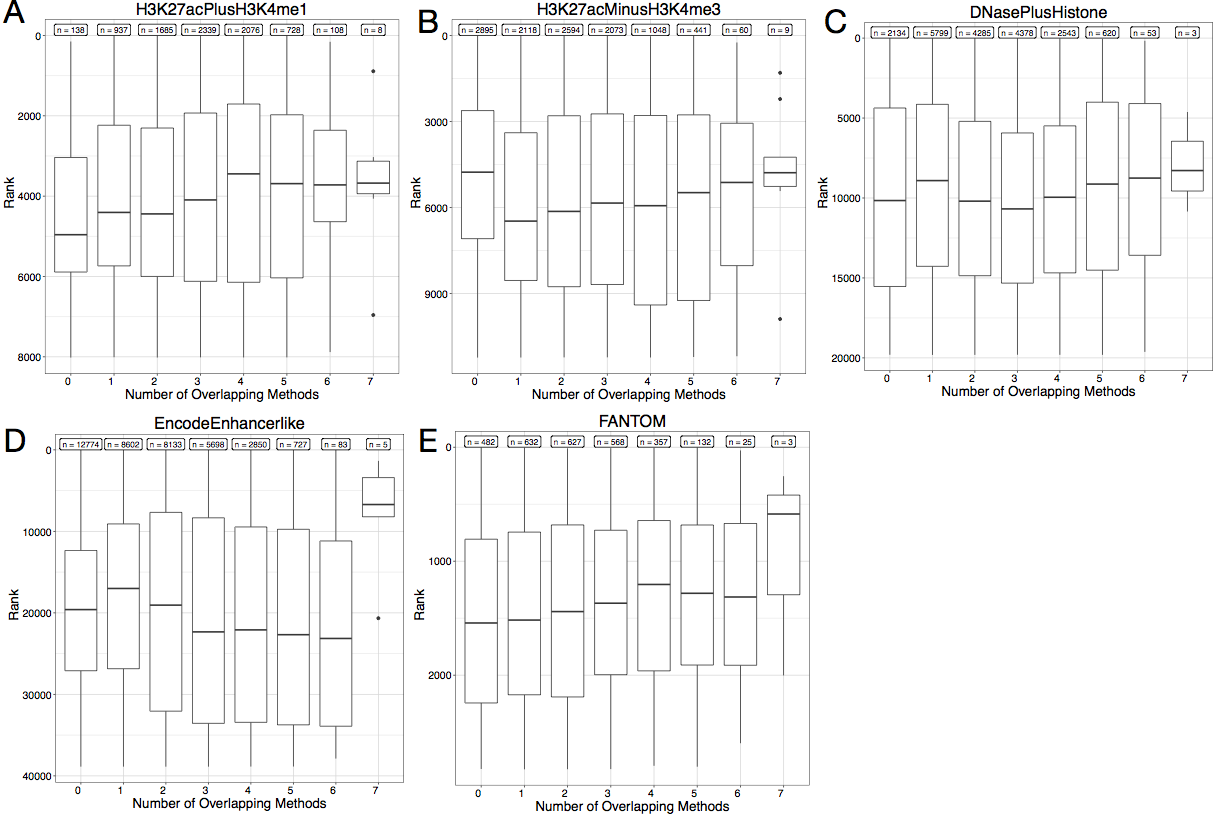


**Figure S17.** Score distributions for Gm12878 enhancer sets are similar between regions identified as enhancers by a single method and those identified by multiple methods: (A) H3K27acPlusH3K4me1, (B) H3K27acMinusH3K4me3, (C) DNasePlusHistone, (D) EncodeEnhancerlike, and (E) FANTOM.

**
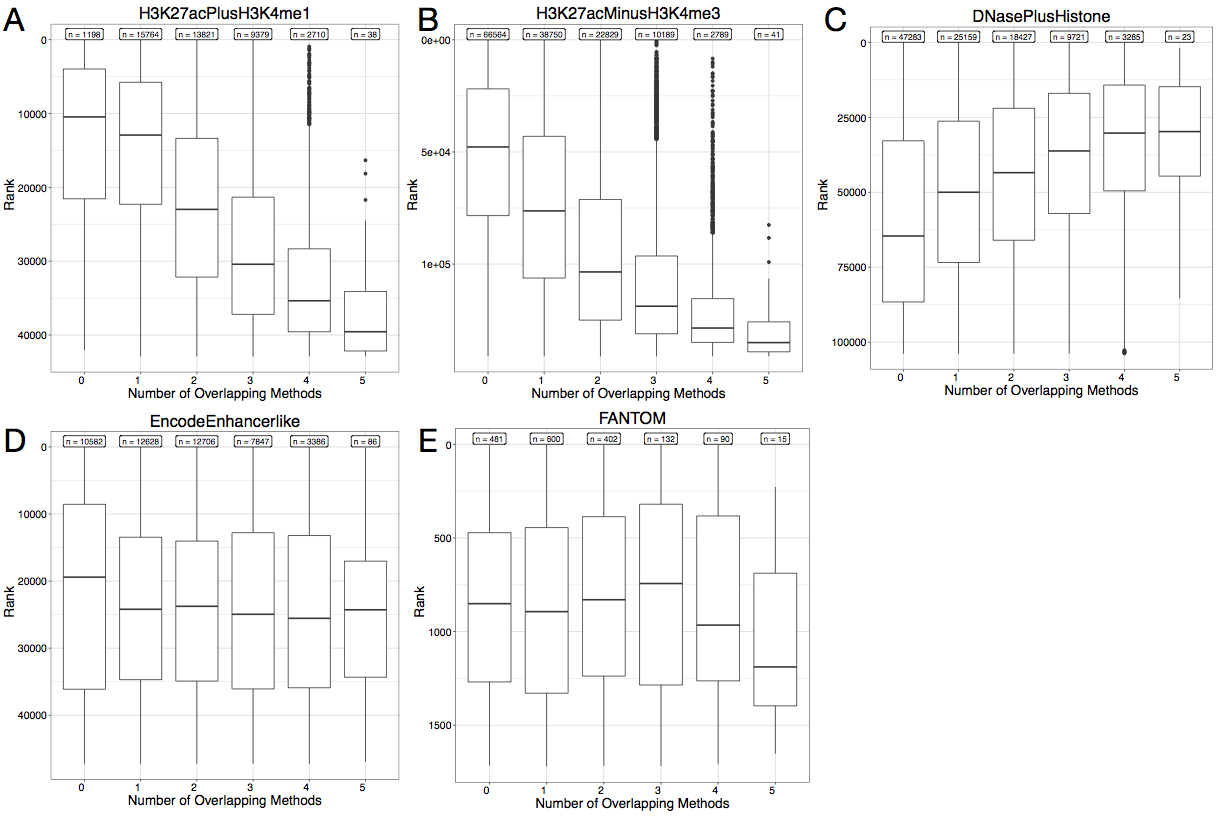
 Figure S18.** Score distributions for heart enhancer sets are similar between regions identified as enhancers by a single method and those identified by multiple methods: (A) H3K27acPlusH3K4me1, (B) H3K27acMinusH3K4me3, (C) DNasePlusHistone, (D) EncodeEnhancerlike, and (E) FANTOM. As in liver enhancer sets (Figure S15), in some cases (A-B) the median score decreases as the regions are more highly shared. This trend may be a result of poor specificity or is potentially a sign of transcription factor binding activity in the region (Ramsey et al. 2010).

**Figure S19.** Enrichment for functional attributes is not significantly different between regions identified as enhancers by a single method and those identified by multiple methods when focusing on the top 100 predictions from each method. For enhancer sets in (A–C) K562, (D–F) Gm12878, (G–I) liver, and (J–L) heart, we consider the top 100 regions per method ranked by confidence or signal scores. This analysis was limited to enhancer sets that could be ranked (DNasePlusHistone, EncodeEnhancerlike, H3K27acPlusH3K4me1, H3K27acMinusH3K4me3, FANTOM). The dotted line represents the level of enrichment expected under a random null distribution; error bars show empirical 95% confidence intervals. This analysis does not include p300 or GRO-cap data for K562 or Gm12878.

 **Figure S20.** Same as Figure S19, but considering the top 500 predictions from each method.

 **Figure S21.** Same as Figure S19, but considering the top 800 predictions from each method.**TABLES**

**Table S1.** The average distance (in bp) to the closest TSS over all enhancers identified by each method in each cellular context.

| **Context** | **Enhancer Set** | **Average Distance to TSS (bp)** |
| --- | --- | --- |
| Gm12878 | EncodeEnhancerlike | 12705.8 |
| Gm12878 | H3K27acPlusH3K4me1 | 25994.1 |
| Gm12878 | Yip12 | 34582.1 |
| Gm12878 | Ho14 | 37669.7 |
| Gm12878 | p300 | 88610.9 |
| Gm12878 | GRO-cap | 30129.3 |
| Gm12878 | FANTOM | 39844.7 |
| Gm12878 | H3K27acMinusH3K4me3 | 40291.6 |
| Gm12878 | DNasePlusHistone | 42544.6 |
| Gm12878 | ChromHMM | 46754.5 |
| Heart | EncodeEnhancerlike | 24725.5 |
| Heart | FANTOM | 34043.2 |
| Heart | ChromHMM | 40694.2 |
| Heart | H3K27acPlusH3K4me1 | 54934.4 |
| Heart | H3K27acMinusH3K4me3 | 63345.5 |
| Heart | VISTA | 67440.9 |
| Heart | DNasePlusHistone | 76044.5 |
| K562 | EncodeEnhancerlike | 13851.6 |
| K562 | H3K27acPlusH3K4me1 | 21384.6 |
| K562 | ChromHMM | 35634.8 |
| K562 | DNasePlusHistone | 36212.6 |
| K562 | Ho14 | 37669.7 |
| K562 | Yip12 | 38038.0 |
| K562 | p300 | 62902.7 |
| K562 | GRO-cap | 35795.7 |
| K562 | H3K27acMinusH3K4me3 | 38040.3 |
| K562 | FANTOM | 45263.2 |
| Liver | EncodeEnhancerlike | 14175.7 |
| Liver | H3K27acPlusH3K4me1 | 32925.0 |
| Liver | FANTOM | 35205.8 |
| Liver | H3K27acMinusH3K4me3 | 43795.9 |
| Liver | ChromHMM | 46308.2 |
| Liver | Villar15 | 35360.8 |
| Liver | DNasePlusHistone | 64407.7 |

**Table S2.** Summary statistics for pairwise percent overlap, both in a base pair and element-wise comparison.

| **Context** | **Comparison Type** | **Minimum** | **Maximum** | **Median** | **Mean** |
| --- | --- | --- | --- | --- | --- |
| K562 | Base-pair | 0.00 | 0.82 | 0.17 | 0.20 |
| K562 | Element | 0.00 | 0.83 | 0.23 | 0.29 |
| Gm12878 | Base-pair | 0.00 | 0.83 | 0.10 | 0.18 |
| Gm12878 | Element | 0.01 | 0.84 | 0.18 | 0.24 |
| Liver | Base-pair | 0.00 | 0.61 | 0.30 | 0.28 |
| Liver | Element | 0.00 | 0.71 | 0.34 | 0.34 |
| Heart | Base-pair | 0.00 | 0.84 | 0.17 | 0.19 |
| Heart | Element | 0.00 | 0.83 | 0.22 | 0.24 |

**Table S3.** Number of observed VISTA heart positive and VISTA negative overlaps for each context and enhancer identification method.

| **Context** | **Enhancer Set** | **Observed VISTA Positive Overlaps** | **Observed VISTA Negative Overlaps** |
| --- | --- | --- | --- |
| Heart | H3K27acPlusH3K4me1 | 19 | 79 |
| Heart | H3K27acMinusH3K4me3 | 36 | 152 |
| Heart | DNasePlusHistone | 25 | 106 |
| Heart | ChromHMM | 71 | 168 |
| Heart | EncodeEnhancerlike | 87 | 179 |
| Heart | FANTOM | 17 | 5 |

**Table S4.** Curated list of relevant GWAS phenotypes for liver (n = 50) and heart (n = 169).

| **Liver Relevant Phenotypes** | **Heart Relevant Phenotypes** |
| --- | --- |
| Aspartate aminotransferase | AR-C124910XX levels in individuals with acute coronary syndromes treated with ticagrelor |
| Autoimmune hepatitis type-1 | Abdominal aortic aneurysm |
| Biliary atresia | Aortic root size |
| Bilirubin levels | Aortic stiffness |
| Bilirubin levels in extreme obesity | Aortic-valve calcification |
| Butyrylcholinesterase levels | Arterial stiffness |
| CYP3A4 enzyme activity | Arterial stiffness (pulse-wave velocity) |
| Drug-induced liver injury | Atrial Septal Defect |
| Drug-induced liver injury (amoxicillin-clavulanate) | Atrial fibrillation |
| Drug-induced liver injury (flucloxacillin) | Atrial fibrillation/atrial flutter |
| Gamma gluatamyl transferase levels | Atrioventricular conduction |
| Gamma gluatamyl transferase levels (interaction with age) | Atrioventricular septal defects in Down syndrome |
| Gamma glutamyl transpeptidase | B-type natriuretic peptide |
| Gaucher disease severity | Blood pressure |
| Hematological and biochemical traits | Blood pressure (age interaction) |
| Hematology traits | Blood pressure (anthropometric measures interaction) |
| Hepatitis | Blood pressure (response to antihypertensive medication) |
| Hepatitis B | Blood pressure (smoking interaction) |
| Hepatitis B (viral clearance) | Blood pressure measurement (cold pressor test) |
| Hepatitis B vaccine response | Blood pressure measurement (high sodium and potassium intervention) |
| Hepatitis C induced liver cirrhosis | Blood pressure measurement (high sodium intervention) |
| Hepatitis C induced liver fibrosis | Blood pressure measurement (low sodium intervention) |
| Hepatocellular carcinoma | Blood pressure response to hydrochlorothiazide in hypertension |
| Hepatocellular carcinoma (hepatitis B virus related) | Blood pressure variability |
| Hepatocellular carcinoma | Brugada syndrome |
| Hepatocellular carcinoma (hepatitis B virus related) | Cardiac Troponin-T levels |
| IFN-related cytopenia | Cardiac hypertrophy |
| Lapatinib-induced hepatotoxicity | Cardiac muscle measurement |
| Lipid levels in hepatitis C treatment | Cardiac repolarization |
| Liver disease in chronic hepatitis B virus infection | Cardiac structure and function |
| Liver enzyme levels | Cardio vascular disease (drug interaction; BB) |
| Liver enzyme levels (alanine transaminase) | Cardioembolic ischaemic stroke |
| Liver enzyme levels (alkaline phosphatase) | Cardiovascular disease (drug interaction, BB) |
| Liver enzyme levels (aspartate transaminase) | Cardiovascular disease (drug interaction, CCB) |
| Liver enzyme levels (gamma-glutamyl transferase) | Cardiovascular disease (drug interaction, diuretics) |
| Lumiracoxib-related liver injury | Cardiovascular disease (drug interaction; ACE) |
| Non-albumin protein levels | Cardiovascular disease risk factors |
| Non-alcoholic fatty liver disease | Cardiovascular heart disease in diabetics |
| Non-alcoholic fatty liver disease histology (AST) | Carotid artery intima media thickness (sex interaction) |
| Non-alcoholic fatty liver disease histology (lobular) | Carotid atherosclerosis (smoking interaction) |
| Non-alcoholic fatty liver disease histology (other) | Carotid atherosclerosis in HIV infection |
| Nonalcoholic fatty liver disease | Carotid intima media thickness |
| Primary biliary cirrhosis | Carotid plaque burden (smoking interaction) |
| Primary sclerosing cholangitis | Cervical artery dissection |
| Response to hepatitis C treatment | Chagas cardiomyopathy in Tripanosoma cruzi seropositivity |
| Response to protease inhibitor treatment in hepatitis c (bilirubin toxicity) | Cholesterol |
| Response to protease inhibitor treatment in hepatitis c (peak serum total bilirubin levels) | Cholesterol and Triglycerides |
| Serum albumin level | Cholesterol, total |
| Serum alkaline phosphatase levels | Circulating vasoactive peptide levels |
| Total bilirubin levels in HIV-1 infection | Clozapine-induced agranulocytosis |
|  | Clozapine-induced cytotoxicity |
|  | Congenital heart disease |
|  | Congenital heart malformation |
|  | Congenital left-sided heart lesions |
|  | Congenital left-sided heart lesions (maternal effect) |
|  | Conotruncal heart defects |
|  | Coronary arterial lesions in patients with Kawasaki disease |
|  | Coronary artery calcification |
|  | Coronary artery calcification (smoking interaction) |
|  | Coronary artery disease |
|  | Coronary artery disease or ischemic stroke |
|  | Coronary artery disease or large artery stroke |
|  | Coronary artery disease-related phenotypes |
|  | Coronary heart disease |
|  | Coronary heart disease event reduction in response to statin therapy (interaction) |
|  | Coronary heart disease in familial hypercholesterolemia |
|  | Coronary restenosis |
|  | Coronary restenosis |
|  | Coronary spasm |
|  | Cystatin C |
|  | Dilated cardiomyopathy |
|  | Drug-induced torsades de pointes |
|  | Echocardiographic traits |
|  | Electrocardiographic conduction measures |
|  | Electrocardiographic traits |
|  | Factor VII |
|  | Factor VII levels |
|  | Factor VIII levels |
|  | Factor XI |
|  | HDL cholesterol |
|  | Heart failure |
|  | Heart rate |
|  | Heart rate variability traits |
|  | Hemostatic factors and hematological phenotypes |
|  | Hypertension |
|  | Hypertension (pulmonary) |
|  | Hypertension (young onset) |
|  | Hypertension risk in short sleep duration |
|  | Hypertrophic cardiomyopathy |
|  | IgE levels |
|  | Ischemic stroke |
|  | LDL (oxidized) |
|  | LDL cholesterol |
|  | LDL cholesterol subfractions |
|  | LDL peak particle diameter (total fat intake interaction) |
|  | Large artery atherosclerosis ischaemic stroke |
|  | Large artery stroke |
|  | Left ventricular mass |
|  | Life threatening arrhythmia |
|  | Lipoprotein-associated phospholipase A2 activity and mass |
|  | Lipoprotein (a) - cholesterol levels |
|  | Lp (a) levels |
|  | Major CVD |
|  | Mitral annular calcification |
|  | Mitral valve prolapse |
|  | Mortality among heart failure patients |
|  | Mortality in heart failure |
|  | Myocardial infarction |
|  | Myocardial infarction (drug interaction; ACE) |
|  | Myocardial infarction (drug interaction; BB) |
|  | Myocardial infarction (drug interaction; CCB) |
|  | Myocardial infarction (drug interaction; diuretics) |
|  | Myocardial infarction (early onset) |
|  | Myocardial infarction in coronary artery disease |
|  | Nonobstructive coronary artery disease |
|  | Oleic acid (18:1n-9) plasma levels |
|  | P wave duration |
|  | PR interval |
|  | PR interval in Tripanosoma cruzi seropositivity |
|  | PR segment |
|  | Palmitic acid (16:0) plasma levels |
|  | Palmitoleic acid (16:1n-7) plasma levels |
|  | Pericardial fat |
|  | Perioperative myocardial infarction in coronary artery bypass surgery |
|  | Peripartum cardiomyopathy |
|  | Plasma cystastin c levels in acute coronary syndrome |
|  | Plasma omega-6 polyunsaturated fatty acid levels (adrenic acid) |
|  | Plasma omega-6 polyunsaturated fatty acid levels (arachidonic acid) |
|  | Plasma omega-6 polyunsaturated fatty acid levels (dihomo-gamma-linolenic acid) |
|  | Plasma omega-6 polyunsaturated fatty acid levels (gamma-linolenic acid) |
|  | Plasma omega-6 polyunsaturated fatty acid levels (linoleic acid) |
|  | Postoperative atrial fibrillation in coronary artery bypass grafting surgery |
|  | Postoperative ventricular dysfunction |
|  | Pulse pressure in young-onset hypertension |
|  | QRS duration |
|  | QRS duration in Tripanosoma cruzi seropositivity |
|  | QT interval |
|  | QT interval (interaction) |
|  | QT interval in Tripanosoma cruzi seropositivity |
|  | RR interval (heart rate) |
|  | Red blood cell count |
|  | Red blood cell fatty acid levels |
|  | Red blood cell traits |
|  | Renal sinus fat |
|  | Response to Dalcetrapib treatment in acute coronary syndrome |
|  | Response to rate control therapy in atrial fibrillation |
|  | Response to statin therapy |
|  | Response to statin therapy (LDL cholesterol subfractions) |
|  | Response to statin therapy (LDL-C) |
|  | Response to statins (LDL cholesterol change) |
|  | Resting heart rate |
|  | Serum dimethylarginine levels (asymmetric/symetric ratio) |
|  | Sick sinus syndrome |
|  | Stearic acid (18:0) plasma levels |
|  | Subclinical atherosclerosis traits (other) |
|  | Sudden cardiac arrest |
|  | Symmetrical dimethylarginine levels |
|  | Tetralogy of Fallot |
|  | Thoracic aortic aneurysms and dissections |
|  | Ticagrelor levels in individuals with acute coronary syndromes treated with ticagrelor |
|  | Triglycerides |
|  | Vascular constriction |
|  | Vein graft stenosis in coronary artery bypass grafting |
|  | Venous thromboembolism |
|  | Venous thromboembolism (SNP x SNP interaction) |
|  | Ventricular conduction |
|  | Ventricular fibrillation |
|  | vWF and FVIII levels |
|  | vWF levels |

**Table S5.** Enrichments for overlap with context-specific SNPs in liver and heart.

| **Context** | **Method** | **Fold Change** | **P Value** |
| --- | --- | --- | --- |
| Liver | H3K27acPlusH3K4me1 | 2.12 | 0.002 |
| Liver | H3K27acMinusH3K4me3 | 2.00 | 0.001 |
| Liver | DNasePlusHistone | 1.74 | 0.035 |
| Liver | ChromHMM | 2.38 | 0.001 |
| Liver | EncodeEnhancerlike | 2.95 | 0.001 |
| Liver | FANTOM | 0.945 | 1.000 |
| Liver | Villar15 | 2.24 | 0.001 |
| Heart | H3K27acPlusH3K4me1 | 2.00 | 0.001 |
| Heart | H3K27acMinusH3K4me3 | 1.87 | 0.001 |
| Heart | DNasePlusHistone | 1.83 | 0.001 |
| Heart | ChromHMM | 2.16 | 0.001 |
| Heart | EncodeEnhancerlike | 2.10 | 0.001 |
| Heart | FANTOM | 2.68 | 0.023 |

**Table S6.** Number of overlapping GWAS SNPs per enhancer identification method and context.

| **Context** | **Enhancer Set** | **Number of GWAS SNPs** | **Number of Context-specific GWAS SNPs** |
| --- | --- | --- | --- |
| K562 | H3K27acPlusH3K4me1 | 269 |  |
| K562 | H3K27acMinusH3K4me3 | 420 |  |
| K562 | DNasePlusHistone | 88 |  |
| K562 | ChromHMM | 1081 |  |
| K562 | EncodeEnhancerlike | 476 |  |
| K562 | Ho14 | 332 |  |
| K562 | Yip12 | 79 |  |
| K562 | p300 | 113 |  |
| K562 | GRO-cap | 63 |  |
| K562 | FANTOM | 2 |  |
| Gm12878 | H3K27acPlusH3K4me1 | 371 |  |
| Gm12878 | H3K27acMinusH3K4me3 | 235 |  |
| Gm12878 | DNasePlusHistone | 161 |  |
| Gm12878 | ChromHMM | 865 |  |
| Gm12878 | EncodeEnhancerlike | 666 |  |
| Gm12878 | Ho14 | 499 |  |
| Gm12878 | Yip12 | 79 |  |
| Gm12878 | p300 | 120 |  |
| Gm12878 | GRO-cap | 68 |  |
| Gm12878 | FANTOM | 24 |  |
| Liver | H3K27acPlusH3K4me1 | 1102 | 25 |
| Liver | H3K27acMinusH3K4me3 | 1658 | 36 |
| Liver | DNasePlusHistone | 654 | 12 |
| Liver | ChromHMM | 1303 | 34 |
| Liver | EncodeEnhancerlike | 1268 | 36 |
| Liver | FANTOM | 3 | 0 |
| Liver | Villar15 | 1203 | 26 |
| Heart | H3K27acPlusH3K4me1 | 644 | 91 |
| Heart | H3K27acMinusH3K4me3 | 1633 | 222 |
| Heart | DNasePlusHistone | 428 | 47 |
| Heart | ChromHMM | 1126 | 155 |
| Heart | EncodeEnhancerlike | 2284 | 302 |
| Heart | FANTOM | 16 | 3 |
| Heart | VISTA | 3 | 0 |

**Table S7.** Enrichments for overlap with context-specific eQTL in liver and heart.

| **Context** | **Method** | **Fold Change** | **P Value** |
| --- | --- | --- | --- |
| Liver | H3K27acPlusH3K4me1 | 1.63 | 0.002 |
| Liver | H3K27acMinusH3K4me3 | 1.62 | 0.001 |
| Liver | DNasePlusHistone | 1.05 | 0.534 |
| Liver | ChromHMM | 1.68 | 0.001 |
| Liver | EncodeEnhancerlike | 1.84 | 0.001 |
| Liver | FANTOM | 0.58 | 0.575 |
| Liver | Villar15 | 1.34 | 0.001 |
| Heart | H3K27acPlusH3K4me1 | 1.42 | 0.001 |
| Heart | H3K27acMinusH3K4me3 | 1.45 | 0.001 |
| Heart | DNasePlusHistone | 1.07 | 0.234 |
| Heart | ChromHMM | 1.35 | 0.001 |
| Heart | EncodeEnhancerlike | 1.76 | 0.001 |
| Heart | FANTOM | 2.49 | 0.006 |

**Table S8.** Number of overlapping GTEx eQTL per enhancer identification method and context.

| **Context** | **Enhancer Set** | **Number of GTEx eQTL** | **Number of Context-specific GTEx eQTL** |
| --- | --- | --- | --- |
| K562 | H3K27acPlusH3K4me1 | 5768 |  |
| K562 | H3K27acMinusH3K4me3 | 8076 |  |
| K562 | DNasePlusHistone | 1561 |  |
| K562 | ChromHMM | 24072 |  |
| K562 | EncodeEnhancerlike | 13142 |  |
| K562 | Ho14 | 6910 |  |
| K562 | Yip12 | 988 |  |
| K562 | p300 | 2062 |  |
| K562 | GRO-cap | 1617 |  |
| K562 | FANTOM | 112 |  |
| Gm12878 | H3K27acPlusH3K4me1 | 7254 |  |
| Gm12878 | H3K27acMinusH3K4me3 | 4653 |  |
| Gm12878 | DNasePlusHistone | 1881 |  |
| Gm12878 | ChromHMM | 14794 |  |
| Gm12878 | EncodeEnhancerlike | 15423 |  |
| Gm12878 | Ho14 | 8672 |  |
| Gm12878 | Yip12 | 988 |  |
| Gm12878 | p300 | 1371 |  |
| Gm12878 | GRO-cap | 1596 |  |
| Gm12878 | FANTOM | 207 |  |
| Liver | H3K27acPlusH3K4me1 | 21426 | 1603 |
| Liver | H3K27acMinusH3K4me3 | 30704 | 2477 |
| Liver | DNasePlusHistone | 9698 | 596 |
| Liver | ChromHMM | 24279 | 2037 |
| Liver | EncodeEnhancerlike | 23962 | 1904 |
| Liver | FANTOM | 95 | 2 |
| Liver | Villar15 | 18648 | 1281 |
| Heart | H3K27acPlusH3K4me1 | 12193 | 3466 |
| Heart | H3K27acMinusH3K4me3 | 32068 | 9165 |
| Heart | DNasePlusHistone | 5380 | 1412 |
| Heart | ChromHMM | 18665 | 5269 |
| Heart | EncodeEnhancerlike | 45916 | 14061 |
| Heart | FANTOM | 162 | 72 |
| Heart | VISTA | 14 | 23 |

**Table S9.** Number of target genes mapped to each enhancer set by JEME. For K562 and Gm12878, p300 and GRO-cap are not included in this mapping.

| **Context** | **Enhancer Set** | **Number of Genes** |
| --- | --- | --- |
| K562 | H3K27acPlusH3K4me1 | 3444 |
| K562 | H3K27acMinusH3K4me3 | 4837 |
| K562 | DNasePlusHistone | 3001 |
| K562 | ChromHMM | 10676 |
| K562 | EncodeEnhancerlike | 10004 |
| K562 | Yip12 | 2754 |
| K562 | Ho14 | 7064 |
| K562 | FANTOM | 3152 |
| Gm12878 | H3K27acPlusH3K4me1 | 4626 |
| Gm12878 | H3K27acMinusH3K4me3 | 3407 |
| Gm12878 | DNasePlusHistone | 5014 |
| Gm12878 | ChromHMM | 10710 |
| Gm12878 | EncodeEnhancerlike | 11303 |
| Gm12878 | Yip12 | 1941 |
| Gm12878 | Ho14 | 8947 |
| Gm12878 | FANTOM | 5352 |
| Liver | H3K27acPlusH3K4me1 | 6871 |
| Liver | H3K27acMinusH3K4me3 | 5964 |
| Liver | DNasePlusHistone | 7176 |
| Liver | ChromHMM | 11788 |
| Liver | EncodeEnhancerlike | 8066 |
| Liver | Villar15 | 3626 |
| Liver | FANTOM | 1796 |
| Heart | H3K27acPlusH3K4me1 | 2121 |
| Heart | H3K27acMinusH3K4me3 | 3940 |
| Heart | DNasePlusHistone | 1771 |
| Heart | ChromHMM | 7144 |
| Heart | EncodeEnhancerlike | 5124 |
| Heart | FANTOM | 1779 |
| Heart | VISTA | 32 |

**Table S11.** Number of enhancers removed by length filtering.

| **Context** | **Enhancer Set** | **Number of Enhancers Removed** |
| --- | --- | --- |
| K562 | H3K27acPlusH3K4me1 | 200 |
| K562 | H3K27acMinusH3K4me3 | 684 |
| K562 | DNasePlusHistone | 220 |
| K562 | ChromHMM | 2117 |
| K562 | EncodeEnhancerlike | 593 |
| K562 | Yip12 | 109 |
| K562 | Ho14 | 765 |
| K562 | p300 | 1178 |
| K562 | GRO-cap | 222 |
| K562 | FANTOM | 21 |
| Gm12878 | H3K27acPlusH3K4me1 | 205 |
| Gm12878 | H3K27acMinusH3K4me3 | 461 |
| Gm12878 | DNasePlusHistone | 400 |
| Gm12878 | ChromHMM | 1300 |
| Gm12878 | EncodeEnhancerlike | 733 |
| Gm12878 | Yip12 | 109 |
| Gm12878 | Ho14 | 766 |
| Gm12878 | p300 | 651 |
| Gm12878 | GRO-cap | 252 |
| Gm12878 | FANTOM | 49 |
| Liver | H3K27acPlusH3K4me1 | 686 |
| Liver | H3K27acMinusH3K4me3 | 2342 |
| Liver | DNasePlusHistone | 3546 |
| Liver | ChromHMM | 1985 |
| Liver | EncodeEnhancerlike | 719 |
| Liver | Villar15 | 225 |
| Liver | FANTOM | 14 |
| Heart | H3K27acPlusH3K4me1 | 590 |
| Heart | H3K27acMinusH3K4me3 | 3908 |
| Heart | DNasePlusHistone | 1693 |
| Heart | ChromHMM | 1978 |
| Heart | EncodeEnhancerlike | 892 |
| Heart | FANTOM | 35 |
| Heart | VISTA | 0 |

**Supplemental References**

Inoue F, Ahituv N. 2015. Decoding enhancers using massively parallel reporter assays. *Genomics* **106**: 159–164. http://dx.doi.org/10.1016/j.ygeno.2015.06.005.

Ramsey SA, Knijnenburg TA, Kennedy KA, Zak DE, Gilchrist M, Gold ES, Johnson CD, Lampano AE, Litvak V, Navarro G, et al. 2010. Genome-wide histone acetylation data improve prediction of mammalian transcription factor binding sites. *Bioinformatics* **26**: 2071–2075.
